# Enhancing Hit Identification in *Mycobacterium tuberculosis* Drug Discovery Using Validated Dual-Event Bayesian Models

**DOI:** 10.1371/journal.pone.0063240

**Published:** 2013-05-07

**Authors:** Sean Ekins, Robert C. Reynolds, Scott G. Franzblau, Baojie Wan, Joel S. Freundlich, Barry A. Bunin

**Affiliations:** 1 Collaborative Drug Discovery, Burlingame, California, United States of America; 2 Collaborations in Chemistry, Fuquay-Varina, North Carolina, United States of America; 3 Southern Research Institute, Birmingham, Alabama, United States of America; 4 Department of Pharmacology & Physiology, UMDNJ – New Jersey Medical School, Newark, New Jersey, United States of America; 5 Institute for Tuberculosis Research, University of Illinois at Chicago, Chicago, Illinois, United States of America; 6 Department of Medicine, Center for Emerging and Reemerging Pathogens, UMDNJ – New Jersey Medical School, Newark, New Jersey, United States of America; Concordia University Wisconsin, United States of America

## Abstract

High-throughput screening (HTS) in whole cells is widely pursued to find compounds active against *Mycobacterium tuberculosis* (*Mtb*) for further development towards new tuberculosis (TB) drugs. Hit rates from these screens, usually conducted at 10 to 25 µM concentrations, typically range from less than 1% to the low single digits. New approaches to increase the efficiency of hit identification are urgently needed to learn from past screening data. The pharmaceutical industry has for many years taken advantage of computational approaches to optimize compound libraries for *in vitro* testing, a practice not fully embraced by academic laboratories in the search for new TB drugs. Adapting these proven approaches, we have recently built and validated Bayesian machine learning models for predicting compounds with activity against *Mtb* based on publicly available large-scale HTS data from the Tuberculosis Antimicrobial Acquisition Coordinating Facility. We now demonstrate the largest prospective validation to date in which we computationally screened 82,403 molecules with these Bayesian models, assayed a total of 550 molecules *in vitro*, and identified 124 actives against *Mtb*. Individual hit rates for the different datasets varied from 15–28%. We have identified several FDA approved and late stage clinical candidate kinase inhibitors with activity against *Mtb* which may represent starting points for further optimization. The computational models developed herein and the commercially available molecules derived from them are now available to any group pursuing *Mtb* drug discovery.

## Introduction

*Mycobacterium tuberculosis* (*Mtb*), the causative agent of tuberculosis (TB), continues to exact a devastating toll on healthcare infrastructure and human life worldwide. *Mtb* infects approximately one-third of the world’s population and kills 1.7–1.8 million people annually [1], on a par with another neglected disease, malaria [2]. While there are very effective treatments for TB, they are neither quick acting nor lacking side effects, resulting in poor patient compliance. In addition, resistance to the available first- and second-line TB drug cocktails is increasing [3], further exacerbated by complicating co-infections with other diseases [4], [5]. There has been a lack of new antibiotic for TB in the last 40 years apart from the recently approved bedaquiline for multidrug resistant TB [6], [7]. There are, however, other promising agents in ongoing clinical trials, although there is an urgent need for back-up and new alternative drugs [8], [9]. Hence, significant investment has been made towards whole-cell phenotypic screening of drug-like small molecule libraries in a search for new compounds that might stem the course of a potential epidemic of totally drug-resistant *Mtb* [5], [10]–[14]. Unfortunately, the hit rate for these costly TB screens in the best cases is in the low single digits (∼1.7–5%) when compound concentrations are 10 µg/ml [13], [14] or 10 µM [12]. It is not uncommon to have hit rates below 1% at concentrations of 14.3 µM [10] and 25 µM [11], as seen elsewhere in high-throughput screening (HTS) and infectious disease drug discovery [15]–[17]. Furthermore, the information from these inefficient and expensive HTS campaigns does not appear to have been used to direct “informed” selection of new libraries in subsequent screens and compound optimization in TB drug discovery. In this regard, virtual screening and computational approaches have been widely adopted in the pharmaceutical industry [18] alongside, or even prior to HTS, to ultimately improve efficiency [19], [20]. There are many computational methods that can assist in identifying compounds with activity against *Mtb* [21]–[24]: these include ligand-based [25] and protein-based [26] methods to identify molecules with ideal physiochemical properties [27].

We have recently conducted an extensive review of the use of computational approaches in TB drug discovery, concluding computational models are employed with little or no integration into the standard TB drug discovery workflow [28]. Furthermore, machine learning and compound classification methods have been infrequently used. These approaches are especially effective for virtual screening of libraries [29], [30]. General classification models such as Bayesian classification models have more recently been tested on datasets of several thousand compounds with activity against *Mtb,* demonstrating classification accuracy greater than 70% [25]. We have also recently reported retrospective Bayesian machine learning model analyses for *Mtb* [22]–[24] using large HTS data sets that were published and made publicly accessible [12], [13]. We observed 8–10 fold enrichment in identifying TB actives in the top scoring molecules. A recently published study [22] applied our TB Bayesian models to datasets published by others and showed four- to ten-fold enrichment factors for the top ranked compounds [22]. For comparison, others have recently described general Bayesian models for antibacterial compounds with 1.5–2 fold enrichments [31]. Such results suggested to us that whole-cell screening data from one laboratory can be used to build machine learning models that appropriately rank compounds screened and identified as *Mtb* hits by others [22]. These previously published models, however, did not account for the cytotoxicity of molecules to mammalian cells lines, e.g. African green monkey (Vero) cells.

Our most recent work has incorporated cytotoxicity data alongside *Mtb* bioactivity data by selecting for relatively non-cytotoxic actives with IC_90_<10 µg/ml (CB2-TAACF [13]) or 10 µM (MLSMR [15]) and a selectivity index (SI) greater than ten. SI was calculated as SI = CC_50_/IC_90_ where CC_50_ is the concentration that resulted in 50% inhibition of Vero cells (CC_50_). In this manner, we have generated Bayesian models [32] with enhanced predictive capability. We prospectively validated these models alongside previous Bayesian models in collaboration with established screening laboratories [32]. We now describe an additional series of three prospective validation experiments using commercially available molecules. Critically, the scale of our prospective validation has increased five-fold from 106 molecules in our recent publication [32] to 550 molecules in this current study that were predicted to be active and relatively non-cytotoxic to cultured Vero cells and experimentally tested. In the process of this evaluation we have further demonstrated the utility of our Bayesian approach to hit discovery and identified valuable starting points for the development of novel antitubercular agents: 124 actives against *Mtb*, including two families built around drug-like heterocyclic cores and several FDA-approved human kinase-targeting drugs. This represents the largest validation of such models against *Mtb* to date.

## Results

We have created and then applied computational TB models, which exploit heterogeneous collections of data. The models are used prospectively to virtually score large libraries of potential antitubercular agents and prioritize them for testing. Empirical assessment of a top-ranked fraction of each library for both antitubercular activity and Vero cell toxicity was then pursued and followed by analyses as to model performance.

### MLSMR Dose Response and Cytotoxicity Model

A dual-event Bayesian model strategy has been recently described which resulted in the MLSMR dose response and cytotoxicity model [32]. The model information is repeated here as we have now made extensive use of it in this study. We selected non-cytotoxic actives as those with IC_90_<10 µΜ and SI>10. This model had a leave-one-out cross-validation receiver operator curve (LOO ROC) value of 0.86 (Table 1). All statistics for this model were equivalent or superior to the previously published MLSMR single point and dose response models (Table S1), which have been extensively validated elsewhere [22]–[24].

**Table 1:** Mean (SD) leave one out and leave out 50%**×**100 cross validation of *Mtb* Bayesian models (ROC = receiver operator characteristic).

Dataset(number of molecules)	Leave one outROC	Leave out 50%×100 External ROC Score	Leave out 50%×100Internal ROC Score	Leave out 50%×100 Concordance	Leave out 50%×100 Specificity	Leave out 50%×100 Sensitivity
MLSMR doseresponse andcytotoxicity (2273)	0.86	0.82±0.02	0.84±0.02	82.61±4.68	83.91±5.48	65.99±7.47
TAACF Kinase single point (23797)	0.89	0.87±0	0.88±0	76.77±2.14	76.49±2.41	81.7±2.96
TAACF Kinase dose response (1248)	0.72	0.65±0.01	0.70±0.01	61.58±1.56	61.85±8.45	61.30±8.24
TAACF Kinase dose response and cytotoxicity (1248)	0.77	0.74±0.02	0.75±0.02	68.67±6.88	69.28±9.84	64.84±12.11

Using the FCFP-6 descriptors we previously identified [32] those substructure descriptors that contribute to the *Mtb* activity (Figure S1) including the oxazole 2-thioether, aryl/heteroaryloxyacetic acid, and quinolone 3-carboxylic acid cores, and those substructure descriptors that are not present in active compounds such as thiazole 2-amides, 2-substituted pyrazoles, 2-substituted benzimidazoles, N-functionalized pyrrolidines, N-arylamides, and 2-substituted pyridines (Figure S2) [32].

### TAACF Kinase Dataset Bayesian Models

The compounds from a library based on kinase inhibitor scaffolds screened through the TAACF was also utilized to construct multiple Bayesian models (Table 1), using the same methodology and validation approach as described previously [22]–[24]. We now describe these for the first time. Using 23,797 compounds with single point *Mtb* screening data we were able to build a Bayesian model with LOO ROC of 0.89. This statistic was stable after leave out 50%**×**100 validation and the model statistics of concordance, specificity and selectivity were >75% (Table 1). From our Bayesian modeling experience, values of >70% for these statistics are acceptable [22]–[24]. Using the FCFP-6 descriptors we identified those substructure descriptors that contribute to *Mtb* activity including 2-substituted 5-membered heterocycles, N-alkylated pyrroles, and imidazoles (Figure S3), and those that are not present in active compounds including imidazolidine diones and aminothienopyridazinones (Figure S4).

When we focused on the subset of 1,248 compounds with dose response data, model statistics decreased (Table 1). ROC values were ∼0.75 and the other statistics dropped to ∼61%, which are low and may be the result of the much smaller dataset being sensitive to the large percentage of data left out for testing compared with the single point model. Although these model statistics are low, the model may still have predictive utility [32]. Using the FCFP-6 descriptors we identified those substructure descriptors that contribute to the *Mtb* activity including 2-substituted 5-membered heterocycles, phenol, fluoroarene, and pyrazolopyrimidine (Figure S5), and those that are not present in active compounds including sulfonamides and nitrobenzene (Figure S6).

When the cytotoxicity data was considered in the active classification (we selected actives as those with IC_90_<10 µg/mL and selectivity SI>10) the ROC increased to >0.75 and all other statistics improved but did not reach levels of the single point model (Table 1). This model was used to evaluate additional compounds. Using the FCFP-6 descriptors we can identify those substructure descriptors that contribute to activity including pyranone and thiophene 2-amides where the amide nitrogen is substituted with an oxazole or oxadiazole (Figure S7), and those that are not present in actives includes 5-alkoxy substituted indole, benzenesulfonamide, pyrazolopyridine, and acylhydrazide (Figure S8).

### Predictions

**Figure 1:**
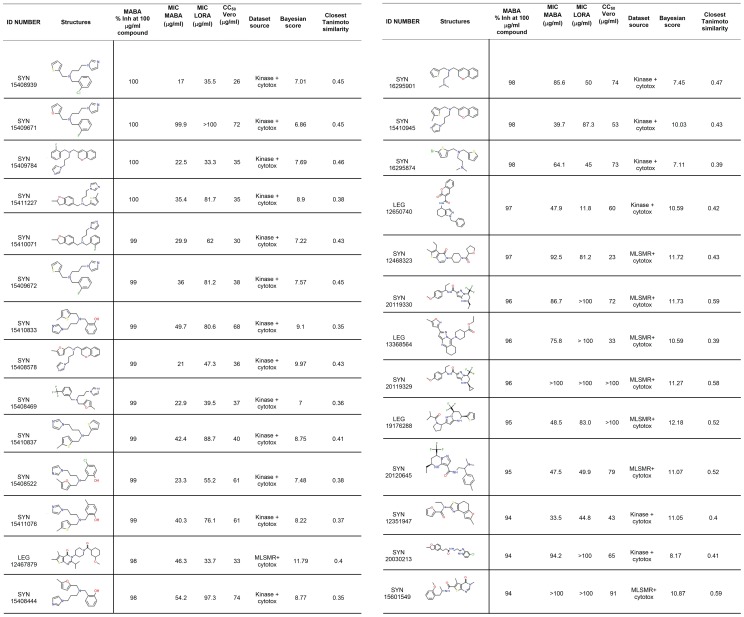
Asinex hits picked with MLSMR dose response and cytotoxicity model and TAACF kinase dose response and cytotoxicity model.More positive numbers from the Bayesian models suggest likely *Mtb* activity.

Both the MLSMR dose response with cytotoxicity and the TAACF kinase dose response with cytotoxicity models were used to screen the Asinex library (N = 25,008), Maybridge library (N = 57,200), and Selleck Chemicals kinase library (N = 194). The libraries were ranked by each model and the top scoring compounds were purchased without any further selection criteria. For the Asinex library, ultimately 94 molecules were selected through scoring with the MLSMR dose response and cytotoxicity model and 88 based on the TAACF kinase dose response and cytotoxicity model. The selected Asinex compounds were purchased and tested at a single concentration. The MLSMR model retrieved 8 hits while the TAACF model correctly predicted 19 hits, where a hit demonstrated >90% inhibition at 100****µg/mL (Figure 1). These represent 8.5% and 21.6% hit rates, respectively, or an overall hit rate of 14.8% when both datasets are combined. For the Maybridge library, 174 total compounds were selected with the same two Bayesian models and 50 molecules had >90% inhibition at 100****µg/mL (Table S2) representing a total 28.7% hit rate. All of the molecules from the Selleck Chemicals kinase library were virtually screened with the MLSMR dose response and cytotoxicity model, the TAACF-CB2 (CB2) dose response and cytotoxicity model (described previously [32]) and the TAACF kinase dose response and cytotoxicity model. Forty-seven molecules had greater than or equal to 90% inhibition of *Mtb* activity at 50 µg/mL (Table S3), representing a total hit rate of 24.2%. The data for all three models can be plotted (Figure S9). Interestingly this shows that the previously published CB2 model [32] has an enrichment of 2.5 fold in the top 5% of compounds at finding compounds (compared to the random hit rate) only based on *Mtb* activity. However, it should also be noted that the TAACF kinase and MLSMR dual-event models performed below random in this analysis.

## Discussion

There is increasing evidence that computational methods can assist in TB research [33]. Our recent efforts have validated the concept that we as a community can more rationally interrogate large public datasets from HTS campaigns that have attempted to find the next antitubercular drug, or its parent hit. Instead of merely taking the “needle in a haystack” approach, we have demonstrated that the data as a whole (actives *and* inactives) can inform us as how to more quickly and inexpensively find actives in future screens. Previous work highlighted a 14% hit rate when applying a single-event Bayesian model and a dual-event model afforded 5/7 hits with an MIC ≤2 µg/ml [32]. The current study greatly expands on these efforts. We are not aware of any other published TB computational studies that have made such extensive prospective predictions using machine learning models followed up by *in vitro* screening for proof of principle. Approaches using docking [28] and inhibitor shape-based methods [34] have recently undergone limited *in vitro* validation important to provide confidence in the outputs. We are observing a shift towards earlier utilization of these more efficient computational methods. The results validate the hypothesis that Bayesian models identify subsets of libraries enriched with actives, through testing a very small percentage (<1%) of the number of compounds usually probed with HTS. For example, the whole-cell screening hit rate in the SRI studies (used to create the Bayesian models) was (∼1.7–5%) [12]–[14]. The Institute for Tuberculosis Research hit rate (Table S5) for screening has depended on compound concentration, assay readout and library type (diversity: 0.67–4.55%; approved drug: 16–21%). In the current study with the computational screening of 82,403 molecules, we assayed 550 molecules and identified 124 actives across commercially available datasets (average hit rate of 22.5%). Such models can clearly select for molecules with whole–cell activity [35], enabling fewer compounds to be tested to find a diverse array of actives. Such an approach may also assist the traditional medicinal chemistry workflow, not only by providing hits more efficiently, but through decreasing the time and cost involved in their evolution to leads and eventually a clinical candidate.

Interestingly, the MLSMR Bayesian model for antitubercular activity previously identified a series of *Mtb* inhibitors with the pyrazolo[1,5-*a*]pyrimidine core [32]. In this study, five of the actives found with the MLSMR dose response and cytotoxicity model (SYN 20119330, SYN 20119329, LEG 19176288, SYN 20120645 and LEG 13368564; Figure 1) maintain the core pyrazole, but have different substitution patterns involving either a fused pyridine or tetrahydropyridine. These may help to expand the structure-activity relationship for this inhibitor class. In addition, we have tested two compounds (SYN22269211 and SYN 22269234) that confirm we can expand on this series (Table S4) and future efforts will specifically examine Bayesian models for hit-to-lead and lead optimization. These are very labor-intensive processes, as typified by the successful evolution of CGI-17341 to the clinical candidate PA-824 over the course of hundreds compounds and many years [36], [37] and the pursuit of next-generation nitroimidazoles by Denny and colleagues [38]–[41].

The TAACF kinase dose response and cytotoxicity model identified novel hits (SYN 15409784, SYN 15411227, SYN 15410071, SYN15409672, SYN15410833, SYN15408578, SYN 15408469, SYN15410837, SYN15408522, SYN 15411076, SYN 15408444 and SYN 15410945 in Figure 1) featuring a tertiary amine nitrogen tethered by three carbons to an imidazole and to two different aromatic moieties each by a methylene. Intriguingly, these molecules share the imidazolylpropylamino functionality of a set of antitubercular hits found via HTS of a commercial kinase-focused library (Table 7 in [14]). Other molecules retrieved by these models appear to be unique (Figure 1). The Bayesian models provide an encouragingly high hit rate for this dataset (14.8%) based on the single concentration MABA data. However, none of these hits displayed a satisfactory SI>10, defined in this case as CC_50_ Vero/MIC MABA, (Figure 1). In contrast, the Maybridge data set (Table S2) showed a higher single concentration MABA hit rate (28.7%) and several apparently selective molecules meeting the SI>10 criteria (BTB05726, BTB14927, HTS 12819, JFD00897, JFD01059, JFD02381, KM02770, and KM03304). It is interesting to note that the MLSMR Bayesian model also correctly rank ordered JFD02381 and JFD02382 which differ by only a methyl group, but have MABA MIC of 5.84 µg/mL and >100 µg/mL, respectively (Table S2).

Surprisingly, many of the known human kinase inhibitors with *Mtb* growth inhibition did not exhibit acceptable SI values of >10. This may reflect the modest antitubercular activity of these small molecules due to comparable binding affinities for kinase ATP binding sites common to targets in both cells [42], [43]. The issues surrounding the concurrent optimizations of *Mtb* kinase inhibition and antitubercular whole-cell efficacy are known [44] and certainly are magnified given the concern over mammalian cell cytotoxicity. Additionally, studies demonstrating the antagonistic effects of human kinase inhibitors on TB infection through, for example, reactivation must be noted [45], [46]. The largest selectivity indices in our study were 6 for XL880 and 3 for NVP-TAE684 and AP24534. Only a few of these kinase inhibitors are approved drugs: lapatinib (breast cancer targeting HER2), sorafenib (renal cell carcinoma, multikinase inhibitor), vandetanib (medullary thyroid cancer, multikinase inhibitor) and regorafenib (metastatic colorectal cancer, VEGFR2-TIE2 inhibitor). While the *in vitro* antitubercular activities of these molecules are likely much poorer than their known human kinase activities, they represent the potential for repurposing [47], [48] and specifically underscore the value of *in silico* repurposing as we have described previously [49], [50]. They may also indicate new targets in *Mtb* to be pursued and significant interest exists in *Mtb* kinases [51], [52]. Based on MABA and LORA MIC values of 5.9 and 5.3 µg/ml, respectively, XL880 may be worthy of follow-up chemistry efforts. This multikinase inhibitor has sub-nanomolar potency versus human c-Met and anti-angiogenesis potential [53]–[55] but has not previously been shown to have activity versus *Mtb*. Others have suggested the approved kinase inhibitor imatinib (gleevec) has antitubercular effects by targeting the host kinases [56]. Direct effects of other compounds on *Mtb* might, therefore, be supplemented by modulating host kinases [57], [58]. Several other studies have screened libraries of kinase inhibitor compounds against whole cell *Mtb* with hit rates of 0.14% [17] and 5% [14] versus cultured *Mtb*. This is the first study to our knowledge in which several of the hits from screening a kinase library have been approved drugs or advanced clinical candidates. Clearly while these compounds target kinases in humans, they also potentiate targets in *Mtb*.

Only one of the three Bayesian models performed better than random in identifying active molecules from the kinase inhibitor dataset. The performance of the TB kinase dose response and cytotoxicity model is perhaps not surprising given its lower validation statistics. The MLSMR dose response and cytotoxicity model, however, performed similarly and displayed much better validation statistics. Clearly other factors are at play such as their differential learning of cytotoxicity from each dataset as well as antitubercular efficacy.

These extensive evaluations combining prospective prediction and *in vitro* testing, suggest Bayesian machine learning models for *Mtb* can identify novel structural classes of antituberculars. While defining actives with both efficacy and selective cytotoxicity may be ideal, our efforts show it is still difficult to achieve this in practice consistently; it is possible in some cases to achieve the desired SI>10 (Table S2). Still more evaluation is warranted to understand how such dual event Bayesian models can provide increased confidence in predictions. We have identified an opportunity for TB researchers to collaboratively use computational models to identify molecules with whole-cell activity and in some cases acceptable mammalian cell cytotoxicity. The weight of evidence we now submit alongside our previous studies [22]–[24], [32], [35] overwhelmingly argues for the inclusion of such computational approaches prior to additional large-scale HTS for *Mtb* based on their ability to identify compounds with whole cell activity alone. We can, thus, focus resources on testing compounds more likely to have favorable activity and promising selectivity. Resources may be saved for more expensive *in vivo* studies and later drug development costs. We now provide this set of 124 hits derived by Bayesian models and validated *in vitro* as a resource to the public to further investigate potential targets and mechanisms by which they are active against *Mtb*. Understanding how we can further optimize these hits and avoid cytotoxicity may lead to new treatments for tuberculosis.

## Materials and Methods

### Ethics Statement

N/A.

### Small Molecules

Small molecules for biological assay were purchased from Asinex Corp. (Winston-Salem, NC), Maybridge/Thermo Fisher Scientific Inc. (Waltham, MA) and Selleck Chemicals (Houston, TX). Compounds were used as supplied from the commercial company. No overt solubility issues were identified.

### CDD Database and SRI Datasets

The development of the CDD TB database (Collaborative Drug Discovery Inc. Burlingame, CA) has been previously described [24]. The Tuberculosis Antimicrobial Acquisition and coordinating Facility (TAACF) and Molecular Libraries Small Molecule Repository (MLSMR) screening datasets [12]–[14] were collected and uploaded in CDD TB from sdf files and mapped to custom protocols [59]. All of the *Mtb* datasets used in model building are available for free public read only access and mining upon registration [60], [61], making them a valuable molecule resource for researchers along with available contextual data on these samples from other non *Mtb* assays. These datasets are also publically available in PubChem [62].

### Machine Learning using Bioactivity and Cytotoxicity Data

We have previously described the generation and validation of Laplacian-corrected Bayesian classifier models [22]–[24] developed with single point screening and dose response data. In this study we have generated Laplacian-corrected Bayesian classifier models using Discovery Studio 2.5.5 [25], [63]–[66]. Molecular function class fingerprints of maximum diameter 6 (FCFP_6) [67], AlogP, molecular weight, number of rotatable bonds, number of rings, number of aromatic rings, number of hydrogen bond acceptors, number of hydrogen bond donors, and molecular fractional polar surface area were calculated from input sdf files using the “calculate molecular properties” protocol to distinguish between compounds that are active against *Mtb* and those that are inactive in this study. Bayesian classifier models with the molecular descriptors described above were built using the “create Bayesian model” protocol and: 1. the MLSMR [12] dose response (IC_90_) and cytotoxicity data for 2,273 compounds (165 active with IC_90_<10 µM and selectivity SI>10 in Vero cells) [32]; 2. the single point screening data for 23,797 compounds from a library based on kinase inhibitor scaffolds (1,308 active; >90% inhibition at 10 µg/ml); 3. half of the maximal inhibitory concentration (IC_50_) dose response data for 1,248 compounds from a library based on kinase inhibitor scaffolds (663 active; IC_50_<5 µg/mL); 4. the IC_90_ and cytotoxicity data for 1,248 compounds from a library based on kinase inhibitor scaffolds (182 active with IC_90_<10 µg/mL and selectivity SI>10 for Vero cells). Each model was validated using leave-one-out (LOO) cross-validation. Each sample was left out one at a time, and a model built using the results of the samples, and that model used to predict the left-out sample. Once all the samples had predictions, a receiver operator curve (ROC) plot was generated, and the cross validated (XV) ROC area under the curve (AUC) calculated (Table 1). All models generated were additionally evaluated by leaving out 50% of the data and rebuilding the model 100 times using a custom protocol for validation, to generate the XV ROC and AUC (Table 1).

### *M. tuberculosis* Assay for Biological Activity

Primary screening and minimum inhibitory concentrations (MIC) against replicating and non-replicating cultures of *Mtb* were determined using the microplate Alamar Blue assay (MABA [68], [69]) (except that 20 µL of 0.6 mM resazurin was used instead of the commercial Alamar Blue reagent) and the low oxygen recovery assay (LORA [70]), respectively. The former was determined against *Mtb* H_37_Rv ATCC 27294 (American Type Culture Collection) following 7 days incubation with test compounds. The latter was determined against low oxygen adapted *Mtb* H_37_Rv *luxAB* carrying a luciferase reporter gene following 10 days incubation under low oxygen followed by 28 hours of normoxic recovery. Both assays were conducted in microplate format in 7H12 medium [69]. For the purposes of this study a hit in the MABA primary screen was defined as achieving at least a 90% reduction in fluorescence relative to untreated controls. The LORA assay was used to determine if any of the hits were also active at low oxygen concentrations. LORA MIC data, was only run once with 8 concentrations and is in line with standard protocols used by this laboratory for many other libraries and studies. The MIC was defined as the lowest concentration producing a reduction of ≥90% in fluorescence (MABA) or luminescence (LORA) relative to untreated controls. Cytotoxicity for Vero cells was determined following 72 hours exposure [69]. Viability was assessed on the basis of cellular conversion of MTS into a soluble formazan product using the Promega CellTiter 96 Aqueous Non-Radioactive Cell Proliferation Assay.

## Supporting Information

Supplemental material is available online. The Bayesian models created in Discovery Studio are available from the authors upon written request.

Figure S1 **MLSMR dose response and cytotoxicity model: good features from FCFP_6.**
(PDF)

Figure S2 **MLSMR dose response and cytotoxicity model: bad features from FCFP_6.**
(PDF)

Figure S3 **TB kinase single point model: good features from FCFP_6.**
(PDF)

Figure S4 **TB kinase single point model: bad features from FCFP_6.**
(PDF)

Figure S5 **TB kinase dose response model: good features from FCFP_6.**
(PDF)

Figure S6 **TB kinase dose response model: bad features from FCFP_6.**
(PDF)

Figure S7 **TB kinase dose response and cytotoxicity model: good features from FCFP_6.**
(PDF)

Figure S8 **TB kinase dose response and cytotoxicity model: bad features from FCFP_6.**
(PDF)

Figure S9 **Results for the 194 compounds tested in the Selleckchem kinase library screened for whole-cell TB activity with Bayesian models.** Random rate is based on the empirical HTS hit rate; MLSMR is based on the MLSMR dose response and cytotoxicity model; CB2 is based on the CB2 dose response and cytotoxicity model [32]. Kinase is based on the MLSMR dose response and cytotoxicity model. Best curve is based on a 100% hit rate.
(PDF)

Table S1 **Mean (SD) leave one out and leave out 50%×100 cross validation of previously published Bayesian models (ROC = receiver operator characteristic) – data from** [**24**] **.**
(PDF)

Table S2 **SRI hits from Maybridge picked using Bayesian dose response and cytotoxicity models.** More positive numbers from the Bayesian models suggest likely activity. SI is the selectivity index calculated by dividing the CC_50_ by either the MIC MABA or MIC LORA. Bold values have an SI greater than 10. Numbers in parentheses are the maximal similarity of the compound to members of the training set for the respective model. Bold is preferred model.
(DOCX)

Table S3 **SRI hits from Selleckchem picked using Bayesian dose response and cytotoxicity models (MLSMR, CB2** [**32**] **and Kinase).** More positive numbers from the Bayesian models suggest likely activity. SI is the selectivity index calculated by dividing the CC_50_ by either the MIC MABA or MIC LORA. Bold values have an SI greater than 10. Numbers in parentheses are the maximal similarity of the compound to members of the training set for the respective model.
(DOCX)

Table S4 **Additional follow up compounds for the pyrazolo[1,5-** ***a*** **]pyrimidine core. SI is the selectivity index calculated by dividing the CC_50_ by either the MIC MABA or MIC LORA.** Bold values have an SI greater than 10.
(DOCX)

Table S5 **Whole cell screening hit rates at Institute for Tuberculosis Research.**
(PDF)
