# Supplementary material for: Enhancing Hit Identification in Mycobacterium tuberculosis Drug Discovery Using Validated Dual-Event Bayesian Models
Source: PLoS One. 2013 May 7;8(5):e63240. doi: 10.1371/journal.pone.0063240 (PMC3647004; doi:10.1371/journal.pone.0063240)
Supplement: Figure S1 — MLSMR dose response and cytotoxicity model: good features from FCFP_6. (PDF) [file pone.0063240.s001.pdf]

Enhancing Hit Identification in *Mycobacterium tuberculosis* Drug Discovery Using Dual-Event Bayesian Models

Sean Ekins[1, 2*], Robert C. Reynolds[3,4], Scott G. Franzblau[5,], Baojie Wan[5] , Joel S. Freundlich[6,7] and Barry A. Bunin[1]

[1]Collaborative Drug Discovery, 1633 Bayshore Highway, Suite 342, Burlingame, CA 94010, USA.

[2]Collaborations in Chemistry, 5616 Hilltop Needmore Road, Fuquay-Varina, NC 27526, USA.

[3]Southern Research Institute, 2000 Ninth Avenue South, Birmingham, AL 35205, USA.

[4]Current address: University of Alabama at Birmingham, College of Arts and Sciences, Department of Chemistry, 1530 3rd Avenue South, Birmingham, Alabama 35294-1240, USA.

[5] Institute for Tuberculosis Research, University of Illinois at Chicago, Chicago, IL 60607, USA.

[6]Department of Medicine, Center for Emerging and Reemerging Pathogens, UMDNJ – New Jersey Medical School, 185 South Orange Avenue Newark, NJ 07103, USA.

[7]Department of Pharmacology & Physiology, UMDNJ – New Jersey Medical School, 185 South Orange Avenue Newark, NJ 07103, USA.

*To whom correspondence should be addressed. (e-mail: ekinssean@yahoo.com)

Running Head: Dual Event Bayesian Models

Figure S1. MLSMR dose response and cytotoxicity model: good features from FCFP_6.

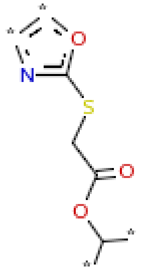<br>G1: -1449793464<br>21 out of 31 good<br>Bayesian Score: 1.896	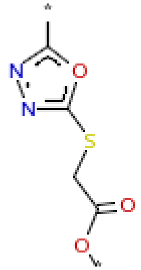<br>G2: -891643182<br>19 out of 27 good<br>Bayesian Score: 1.895	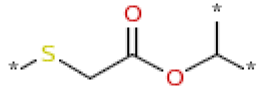<br>G3: -60038150<br>49 out of 89 good<br>Bayesian Score: 1.882	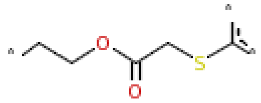<br>G4: 1670875748<br>24 out of 38 good<br>Bayesian Score: 1.877	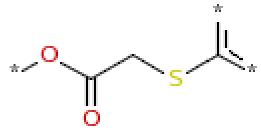<br>G5: 363977085<br>49 out of 92 good<br>Bayesian Score: 1.853
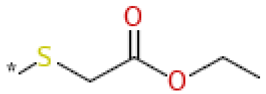<br>G6: 854938188<br>21 out of 36 good<br>Bayesian Score: 1.789	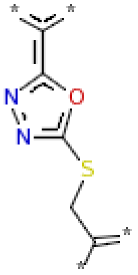<br>G7: -1387481105<br>25 out of 47 good<br>Bayesian Score: 1.755	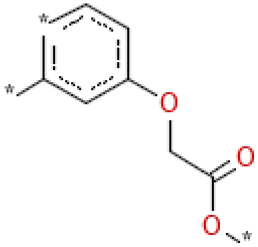<br>G8: 1209850658<br>12 out of 17 good<br>Bayesian Score: 1.748	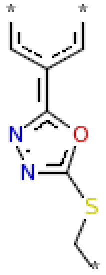<br>G9: -1700730929<br>24 out of 46 good<br>Bayesian Score: 1.733	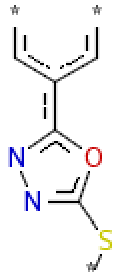<br>G10: 1476473713<br>24 out of 47 good<br>Bayesian Score: 1.716

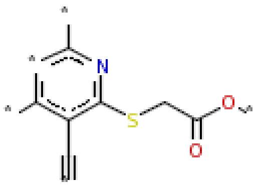<br>G11: -1943087851<br>9 out of 11 good<br>Bayesian Score: 1.705	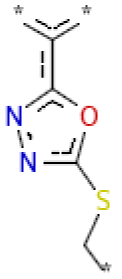<br>G12: 1941608633<br>25 out of 51 good<br>Bayesian Score: 1.691	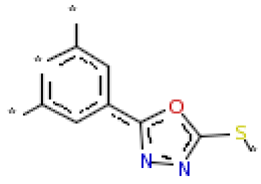<br>G13: -180488964<br>19 out of 37 good<br>Bayesian Score: 1.674	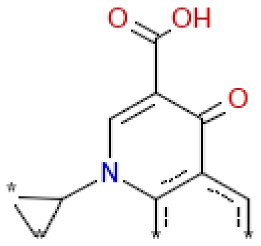<br>G14: 1048446020<br>7 out of 7 good<br>Bayesian Score: 1.660	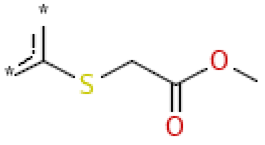<br>G15: 1669687439<br>13 out of 23 good<br>Bayesian Score: 1.642
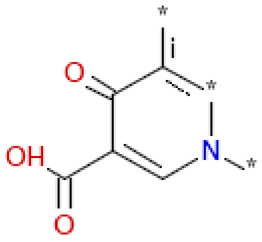<br>G16: 173580544<br>8 out of 10 good<br>Bayesian Score: 1.641	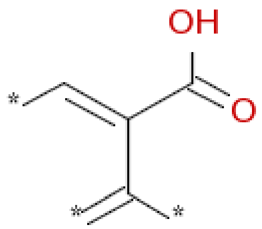<br>G17: 1393614142<br>8 out of 10 good<br>Bayesian Score: 1.641	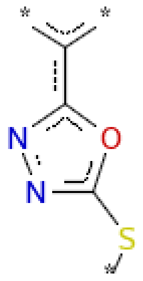<br>G18: -1048017265<br>25 out of 55 good<br>Bayesian Score: 1.631	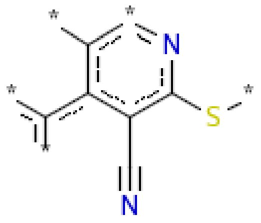<br>G19: 715653355<br>7 out of 8 good<br>Bayesian Score: 1.613	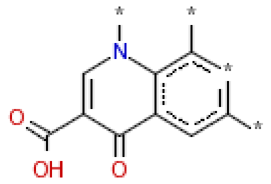<br>G20: 192299924<br>7 out of 8 good<br>Bayesian Score: 1.613
