# Supplementary material for: Enhancing Hit Identification in Mycobacterium tuberculosis Drug Discovery Using Validated Dual-Event Bayesian Models
Source: PLoS One. 2013 May 7;8(5):e63240. doi: 10.1371/journal.pone.0063240 (PMC3647004; doi:10.1371/journal.pone.0063240)
Supplement: Figure S2 — MLSMR dose response and cytotoxicity model: bad features from FCFP_6. (PDF) [file pone.0063240.s002.pdf]

Enhancing Hit Identification in *Mycobacterium tuberculosis* Drug Discovery Using Dual-Event Bayesian Models

Sean Ekins[1, 2*], Robert C. Reynolds[3,4], Scott G. Franzblau[5,], Baojie Wan[5], Joel S. Freundlich[6,7] and Barry A. Bunin[1]

[1]Collaborative Drug Discovery, 1633 Bayshore Highway, Suite 342, Burlingame, CA 94010, USA.

[2]Collaborations in Chemistry, 5616 Hilltop Needmore Road, Fuquay-Varina, NC 27526, USA.

[3]Southern Research Institute, 2000 Ninth Avenue South, Birmingham, AL 35205, USA.

[4]Current address: University of Alabama at Birmingham, College of Arts and Sciences, Department of Chemistry, 1530 3rd Avenue South, Birmingham, Alabama 35294-1240, USA.

[5] Institute for Tuberculosis Research, University of Illinois at Chicago, Chicago, IL 60607, USA.

[6]Department of Medicine, Center for Emerging and Reemerging Pathogens, UMDNJ – New Jersey Medical School, 185 South Orange Avenue Newark, NJ 07103, USA.

[7]Department of Pharmacology & Physiology, UMDNJ – New Jersey Medical School, 185 South Orange Avenue Newark, NJ 07103, USA.

*To whom correspondence should be addressed. (e-mail: ekinssean@yahoo.com)

Running Head: Dual Event Bayesian Models

Figure S2. MLSMR dose response and cytotoxicity model: bad features from FCFP_6.

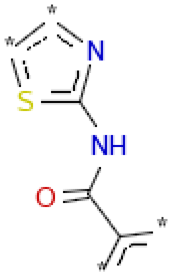	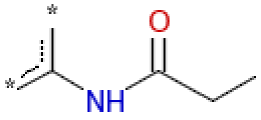	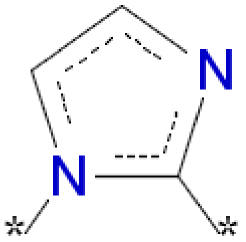	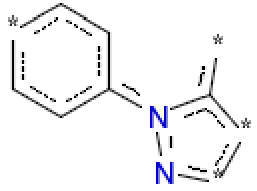	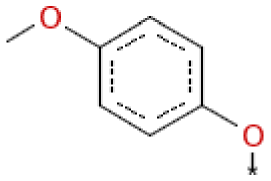
B1: 741645840<br>0 out of 102 good<br>Bayesian Score: -2.150	B2: -1944142687<br>0 out of 74 good<br>Bayesian Score: -1.872	B3: -1956934524<br>0 out of 74 good<br>Bayesian Score: -1.872	B4: -2022638555<br>0 out of 71 good<br>Bayesian Score: -1.837	B5: 356782498<br>0 out of 68 good<br>Bayesian Score: -1.801
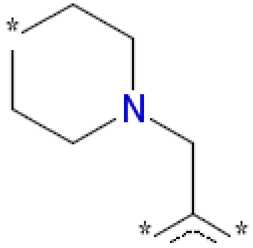	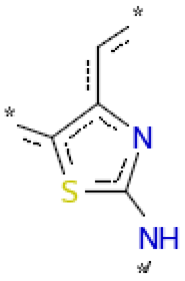	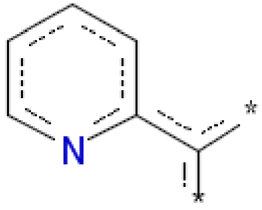	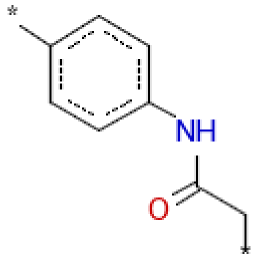	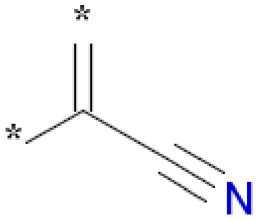
B6: 373464202<br>0 out of 62 good<br>Bayesian Score: -1.724	B7: -1771983677<br>0 out of 62 good<br>Bayesian Score: -1.724	B8: -1757378719<br>0 out of 62 good<br>Bayesian Score: -1.724	B9: -451043714<br>1 out of 131 good<br>Bayesian Score: -1.681	B10: -1277879912<br>0 out of 58 good<br>Bayesian Score: -1.670

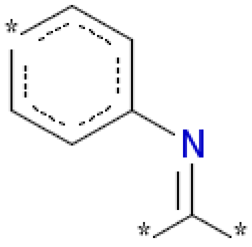<br>B11: -1699460258<br>0 out of 56 good<br>Bayesian Score: -1.642	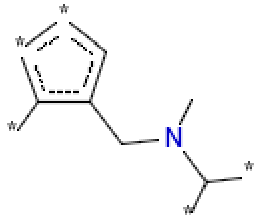<br>B12: 1848358530<br>0 out of 55 good<br>Bayesian Score: -1.627	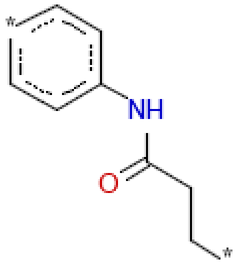<br>B13: 1904392374<br>0 out of 54 good<br>Bayesian Score: -1.612	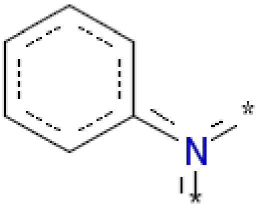<br>B14: -2094771862<br>0 out of 54 good<br>Bayesian Score: -1.612	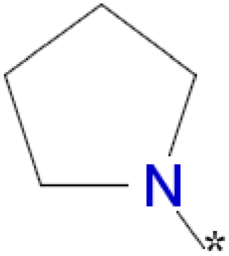<br>B15: 240509252<br>0 out of 51 good<br>Bayesian Score: -1.567
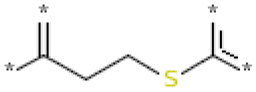<br>B16: 368675221<br>0 out of 50 good<br>Bayesian Score: -1.551	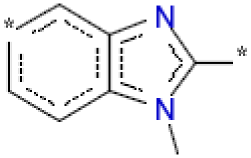<br>B17: 1135306861<br>0 out of 49 good<br>Bayesian Score: -1.535	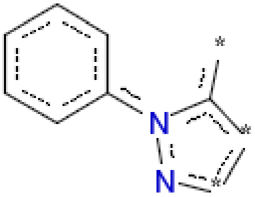<br>B18: -2129165368<br>0 out of 48 good<br>Bayesian Score: -1.519	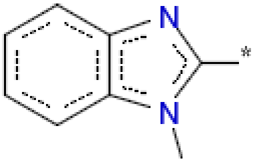<br>B19: -1775219197<br>0 out of 46 good<br>Bayesian Score: -1.486	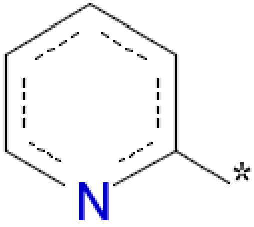<br>B20: 2117749172<br>1 out of 104 good<br>Bayesian Score: -1.474
