# Supplementary material for: Enhancing Hit Identification in Mycobacterium tuberculosis Drug Discovery Using Validated Dual-Event Bayesian Models
Source: PLoS One. 2013 May 7;8(5):e63240. doi: 10.1371/journal.pone.0063240 (PMC3647004; doi:10.1371/journal.pone.0063240)
Supplement: Figure S3 — TB kinase single point model: good features from FCFP_6. (PDF) [file pone.0063240.s003.pdf]

Enhancing Hit Identification in *Mycobacterium tuberculosis* Drug Discovery Using Dual-Event Bayesian Models

Sean Ekins[1, 2*], Robert C. Reynolds[3,4], Scott G. Franzblau[5,], Baojie Wan[5] , Joel S. Freundlich[6,7] and Barry A. Bunin[1]

[1]Collaborative Drug Discovery, 1633 Bayshore Highway, Suite 342, Burlingame, CA 94010, USA.

[2]Collaborations in Chemistry, 5616 Hilltop Needmore Road, Fuquay-Varina, NC 27526, USA.

[3]Southern Research Institute, 2000 Ninth Avenue South, Birmingham, AL 35205, USA.

[4]Current address: University of Alabama at Birmingham, College of Arts and Sciences, Department of Chemistry, 1530 3rd Avenue South, Birmingham, Alabama 35294-1240, USA.

[5] Institute for Tuberculosis Research, University of Illinois at Chicago, Chicago, IL 60607, USA.

[6]Department of Medicine, Center for Emerging and Reemerging Pathogens, UMDNJ – New Jersey Medical School, 185 South Orange Avenue Newark, NJ 07103, USA.

[7]Department of Pharmacology & Physiology, UMDNJ – New Jersey Medical School, 185 South Orange Avenue Newark, NJ 07103, USA.

*To whom correspondence should be addressed. (e-mail: ekinssean@yahoo.com)

Running Head: Dual Event Bayesian Models

Figure S3. TB kinase single point model: good features from FCFP_6.

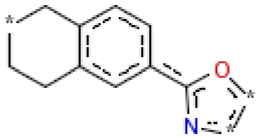<br>G1: 2117846299<br>46 out of 51 good<br>Bayesian Score: 2.536	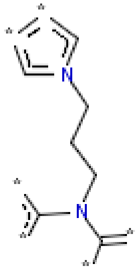<br>G2: -1748509072<br>60 out of 75 good<br>Bayesian Score: 2.501	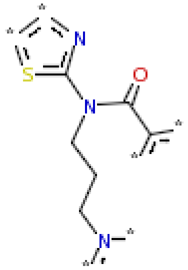<br>G3: 1507383929<br>44 out of 52 good<br>Bayesian Score: 2.478	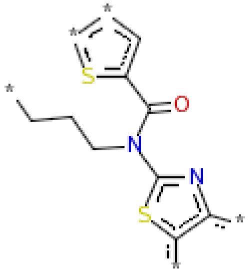<br>G4: 1709560400<br>27 out of 28 good<br>Bayesian Score: 2.418	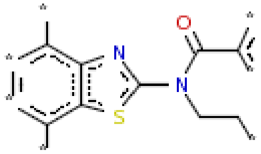<br>G5: 1151340232<br>45 out of 59 good<br>Bayesian Score: 2.406
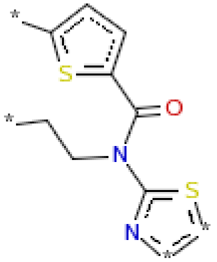<br>G6: -465936875<br>27 out of 29 good<br>Bayesian Score: 2.397	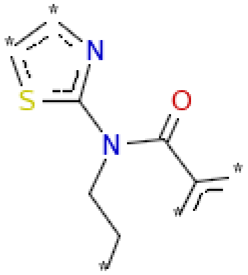<br>G7: -609539162<br>45 out of 63 good<br>Bayesian Score: 2.355	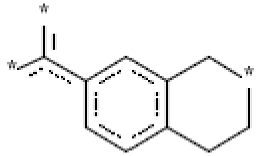<br>G8: -1946821836<br>48 out of 76 good<br>Bayesian Score: 2.271	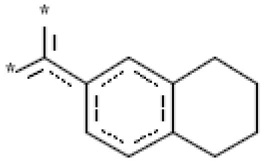<br>G9: 9642716<br>48 out of 76 good<br>Bayesian Score: 2.271	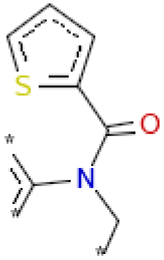<br>G10: 1662717112<br>27 out of 36 good<br>Bayesian Score: 2.260

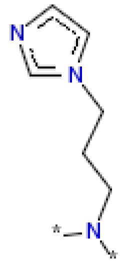<br>G11: -1298621177<br>60 out of 105 good<br>Bayesian Score: 2.223	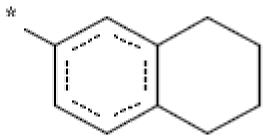<br>G12: -936579330<br>61 out of 108 good<br>Bayesian Score: 2.215	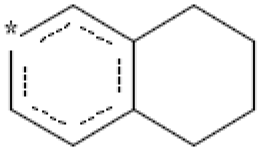<br>G13: -1248094339<br>61 out of 110 good<br>Bayesian Score: 2.200	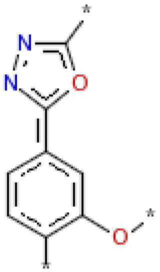<br>G14: -1651220302<br>66 out of 126 good<br>Bayesian Score: 2.160	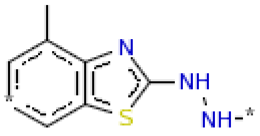<br>G15: -1633399590<br>28 out of 45 good<br>Bayesian Score: 2.143
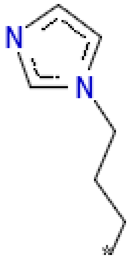<br>G16: -1461049343<br>62 out of 122 good<br>Bayesian Score: 2.127	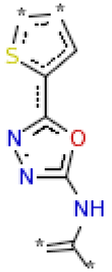<br>G17: 1409643723<br>79 out of 160 good<br>Bayesian Score: 2.126	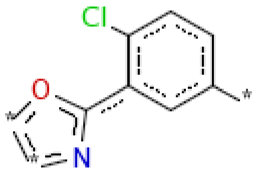<br>G18: -219979617<br>38 out of 69 good<br>Bayesian Score: 2.120	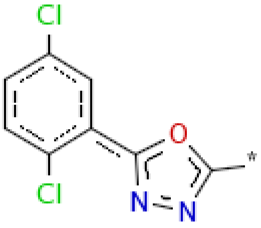<br>G19: -128762487<br>38 out of 69 good<br>Bayesian Score: 2.120	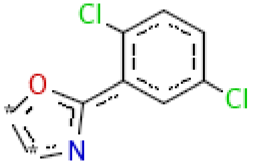<br>G20: 1935545764<br>38 out of 69 good<br>Bayesian Score: 2.120
