# Supplementary material for: Enhancing Hit Identification in Mycobacterium tuberculosis Drug Discovery Using Validated Dual-Event Bayesian Models
Source: PLoS One. 2013 May 7;8(5):e63240. doi: 10.1371/journal.pone.0063240 (PMC3647004; doi:10.1371/journal.pone.0063240)
Supplement: Figure S4 — TB kinase single point model: bad features from FCFP_6. (PDF) [file pone.0063240.s004.pdf]

Enhancing Hit Identification in *Mycobacterium tuberculosis* Drug Discovery Using Dual-Event Bayesian Models

Sean Ekins[1, 2*], Robert C. Reynolds[3,4], Scott G. Franzblau[5,], Baojie Wan[5] , Joel S. Freundlich[6,7] and Barry A. Bunin[1]

[1]Collaborative Drug Discovery, 1633 Bayshore Highway, Suite 342, Burlingame, CA 94010, USA.

[2]Collaborations in Chemistry, 5616 Hilltop Needmore Road, Fuquay-Varina, NC 27526, USA.

[3]Southern Research Institute, 2000 Ninth Avenue South, Birmingham, AL 35205, USA.

[4]Current address: University of Alabama at Birmingham, College of Arts and Sciences, Department of Chemistry, 1530 3rd Avenue South, Birmingham, Alabama 35294-1240, USA.

[5] Institute for Tuberculosis Research, University of Illinois at Chicago, Chicago, IL 60607, USA.

[6]Department of Medicine, Center for Emerging and Reemerging Pathogens, UMDNJ – New Jersey Medical School, 185 South Orange Avenue Newark, NJ 07103, USA.

[7]Department of Pharmacology & Physiology, UMDNJ – New Jersey Medical School, 185 South Orange Avenue Newark, NJ 07103, USA.

*To whom correspondence should be addressed. (e-mail: ekinssean@yahoo.com)

Running Head: Dual Event Bayesian Models

Figure S4. TB kinase single point model: bad features from FCFP_6.

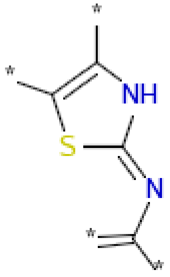<br>B1: 78996860<br>0 out of 649 good<br>Bayesian Score: -3.574	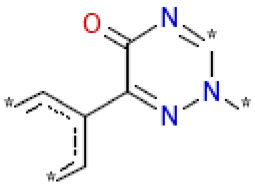<br>B2: 1780681940<br>0 out of 609 good<br>Bayesian Score: -3.512	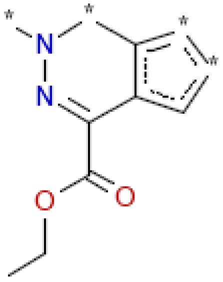<br>B3: -186241159<br>0 out of 608 good<br>Bayesian Score: -3.510	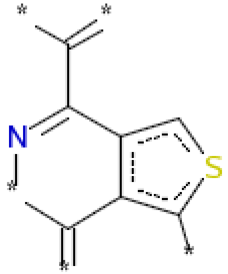<br>B4: -163956986<br>0 out of 608 good<br>Bayesian Score: -3.510	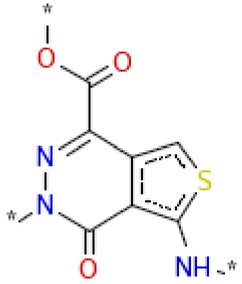<br>B5: 1488194790<br>0 out of 608 good<br>Bayesian Score: -3.510
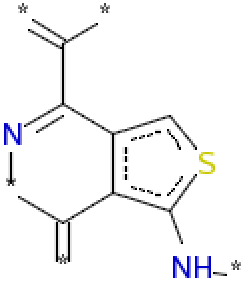<br>B6: 1642535433<br>0 out of 608 good<br>Bayesian Score: -3.510	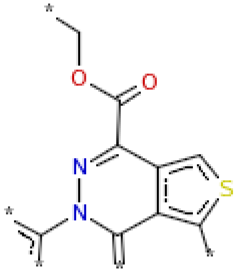<br>B7: 792169024<br>0 out of 608 good<br>Bayesian Score: -3.510	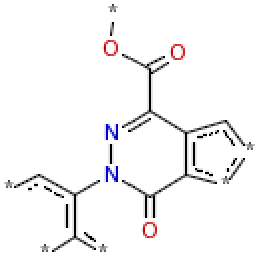<br>B8: 1040673898<br>0 out of 608 good<br>Bayesian Score: -3.510	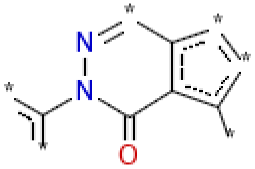<br>B9: 389259921<br>0 out of 608 good<br>Bayesian Score: -3.510	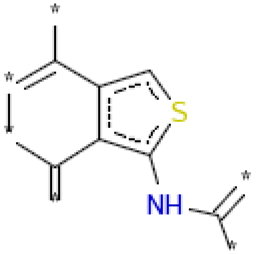<br>B10: 229482314<br>0 out of 608 good<br>Bayesian Score: -3.510

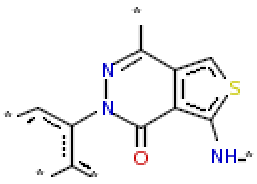 B11: 10587343<br>0 out of 608 good<br>Bayesian Score: -3.510	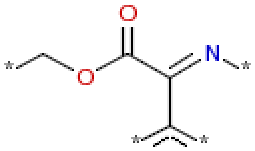 B12: 909560912<br>0 out of 608 good<br>Bayesian Score: -3.510	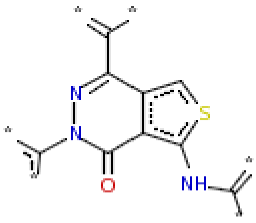 B13: -88401076<br>0 out of 608 good<br>Bayesian Score: -3.510	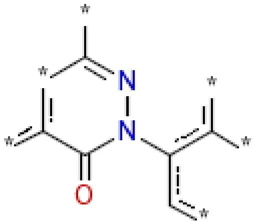 B14: -237342290<br>0 out of 608 good<br>Bayesian Score: -3.510	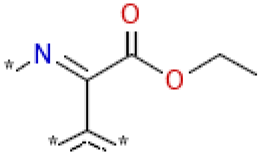 B15: 859865998<br>0 out of 608 good<br>Bayesian Score: -3.510
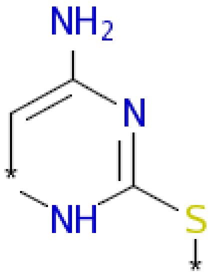 B16: -223337671<br>0 out of 605 good<br>Bayesian Score: -3.505	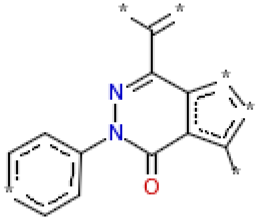 B17: -118187393<br>0 out of 605 good<br>Bayesian Score: -3.505	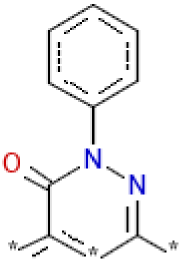 B18: 1561760743<br>0 out of 590 good<br>Bayesian Score: -3.481	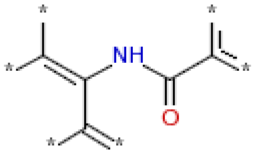 B19: 150315108<br>0 out of 515 good<br>Bayesian Score: -3.350	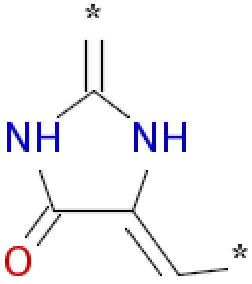 B20: -971446717<br>0 out of 513 good<br>Bayesian Score: -3.346
