# Supplementary material for: Enhancing Hit Identification in Mycobacterium tuberculosis Drug Discovery Using Validated Dual-Event Bayesian Models
Source: PLoS One. 2013 May 7;8(5):e63240. doi: 10.1371/journal.pone.0063240 (PMC3647004; doi:10.1371/journal.pone.0063240)
Supplement: Figure S5 — TB kinase dose response model: good features from FCFP_6. (PDF) [file pone.0063240.s005.pdf]

Enhancing Hit Identification in *Mycobacterium tuberculosis* Drug Discovery Using Dual-Event Bayesian Models

Sean Ekins[1, 2*], Robert C. Reynolds[3,4], Scott G. Franzblau[5,], Baojie Wan[5] , Joel S. Freundlich[6,7] and Barry A. Bunin[1]

[1]Collaborative Drug Discovery, 1633 Bayshore Highway, Suite 342, Burlingame, CA 94010, USA.

[2]Collaborations in Chemistry, 5616 Hilltop Needmore Road, Fuquay-Varina, NC 27526, USA.

[3]Southern Research Institute, 2000 Ninth Avenue South, Birmingham, AL 35205, USA.

[4]Current address: University of Alabama at Birmingham, College of Arts and Sciences, Department of Chemistry, 1530 3rd Avenue South, Birmingham, Alabama 35294-1240, USA.

[5] Institute for Tuberculosis Research, University of Illinois at Chicago, Chicago, IL 60607, USA.

[6]Department of Medicine, Center for Emerging and Reemerging Pathogens, UMDNJ – New Jersey Medical School, 185 South Orange Avenue Newark, NJ 07103, USA.

[7]Department of Pharmacology & Physiology, UMDNJ – New Jersey Medical School, 185 South Orange Avenue Newark, NJ 07103, USA.

[*]To whom correspondence should be addressed. (e-mail: ekinssean@yahoo.com)

Running Head: Dual Event Bayesian Models

Figure S5. TB kinase dose response model: good features from FCFP_6.

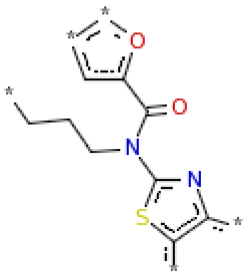<br>G1: 1709560400<br>26 out of 27 good<br>Bayesian Score: 0.574	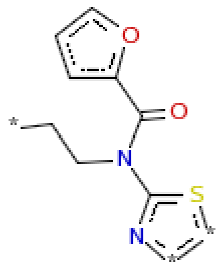<br>G2: -465936875<br>26 out of 27 good<br>Bayesian Score: 0.574	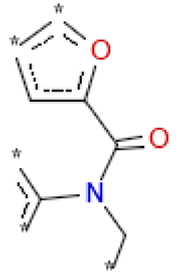<br>G3: 1884734655<br>26 out of 27 good<br>Bayesian Score: 0.574	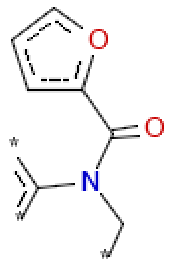<br>G4: 1662717112<br>25 out of 26 good<br>Bayesian Score: 0.572	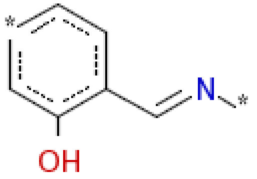<br>G5: 1972951585<br>11 out of 11 good<br>Bayesian Score: 0.570
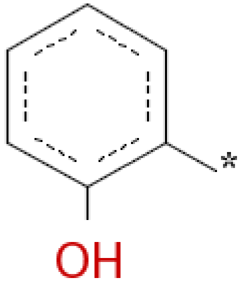<br>G6: 667078692<br>11 out of 11 good<br>Bayesian Score: 0.570	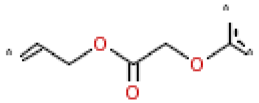<br>G7: 1670875748<br>9 out of 9 good<br>Bayesian Score: 0.556	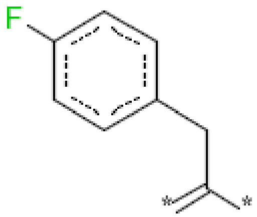<br>G8: 1979799016<br>9 out of 9 good<br>Bayesian Score: 0.556	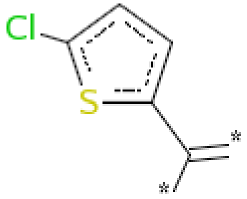<br>G9: -1459497616<br>18 out of 19 good<br>Bayesian Score: 0.547	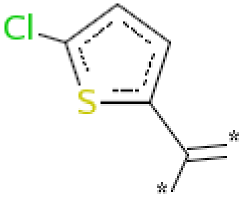<br>G10: -1319897555<br>18 out of 19 good<br>Bayesian Score: 0.547

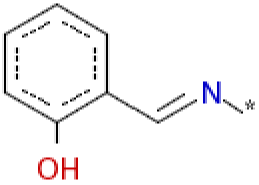<br>G11: 573330716<br>8 out of 8 good<br>Bayesian Score: 0.547	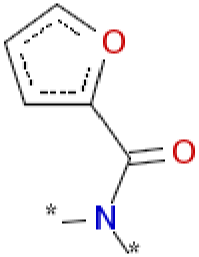<br>G12: 387255155<br>27 out of 29 good<br>Bayesian Score: 0.544	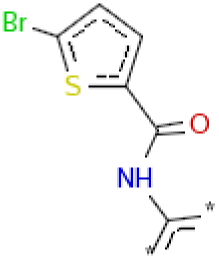<br>G13: -1440282487<br>17 out of 18 good<br>Bayesian Score: 0.542	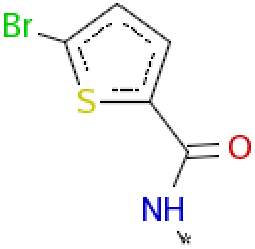<br>G14: 747969531<br>17 out of 18 good<br>Bayesian Score: 0.542	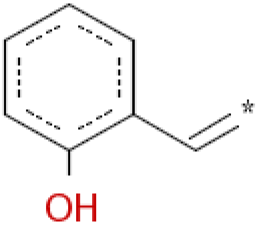<br>G15: -309153329<br>7 out of 7 good<br>Bayesian Score: 0.536
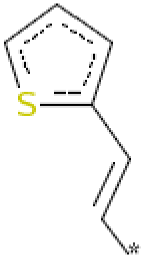<br>G16: 1294872042<br>15 out of 16 good<br>Bayesian Score: 0.530	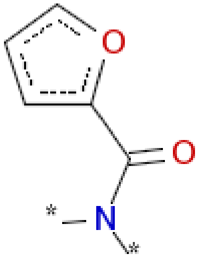<br>G17: -1201618245<br>30 out of 33 good<br>Bayesian Score: 0.524	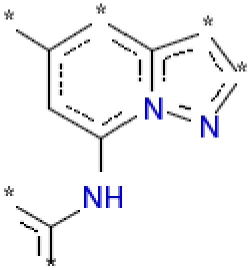<br>G18: 200492893<br>6 out of 6 good<br>Bayesian Score: 0.521	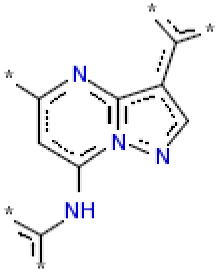<br>G19: -698859809<br>6 out of 6 good<br>Bayesian Score: 0.521	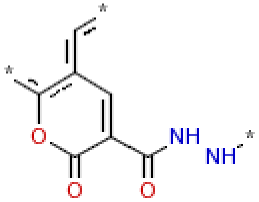<br>G20: 1825191782<br>6 out of 6 good<br>Bayesian Score: 0.521
