# Supplementary material for: Enhancing Hit Identification in Mycobacterium tuberculosis Drug Discovery Using Validated Dual-Event Bayesian Models
Source: PLoS One. 2013 May 7;8(5):e63240. doi: 10.1371/journal.pone.0063240 (PMC3647004; doi:10.1371/journal.pone.0063240)
Supplement: Figure S6 — TB kinase dose response model: bad features from FCFP_6. (PDF) [file pone.0063240.s006.pdf]

Enhancing Hit Identification in *Mycobacterium tuberculosis* Drug Discovery Using Dual-Event Bayesian Models

Sean Ekins[1, 2*], Robert C. Reynolds[3,4], Scott G. Franzblau[5,], Baojie Wan[5] , Joel S. Freundlich[6,7] and Barry A. Bunin[1]

[1]Collaborative Drug Discovery, 1633 Bayshore Highway, Suite 342, Burlingame, CA 94010, USA.

[2]Collaborations in Chemistry, 5616 Hilltop Needmore Road, Fuquay-Varina, NC 27526, USA.

[3]Southern Research Institute, 2000 Ninth Avenue South, Birmingham, AL 35205, USA.

[4]Current address: University of Alabama at Birmingham, College of Arts and Sciences, Department of Chemistry, 1530 3rd Avenue South, Birmingham, Alabama 35294-1240, USA.

[5] Institute for Tuberculosis Research, University of Illinois at Chicago, Chicago, IL 60607, USA.

[6]Department of Medicine, Center for Emerging and Reemerging Pathogens, UMDNJ – New Jersey Medical School, 185 South Orange Avenue Newark, NJ 07103, USA.

[7]Department of Pharmacology & Physiology, UMDNJ – New Jersey Medical School, 185 South Orange Avenue Newark, NJ 07103, USA.

*To whom correspondence should be addressed. (e-mail: ekinssean@yahoo.com)

Running Head: Dual Event Bayesian Models

Figure S6. TB kinase dose response model: bad features from FCFP_6.

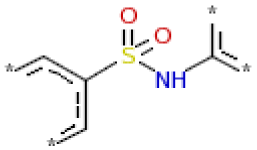<br>B1: 1183237506<br>0 out of 9 good<br>Bayesian Score: -1.747	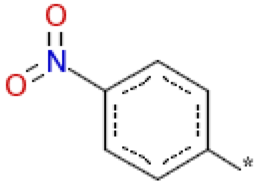<br>B2: 984117435<br>0 out of 8 good<br>Bayesian Score: -1.650	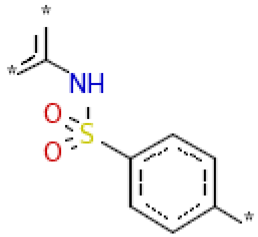<br>B3: -1482227438<br>0 out of 8 good<br>Bayesian Score: -1.650	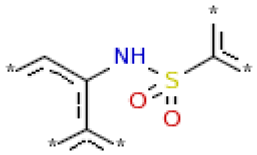<br>B4: 1176920513<br>0 out of 7 good<br>Bayesian Score: -1.544	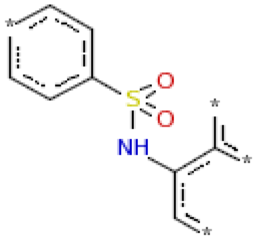<br>B5: 1980808403<br>0 out of 7 good<br>Bayesian Score: -1.544
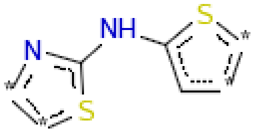<br>B6: 1122517666<br>0 out of 7 good<br>Bayesian Score: -1.544	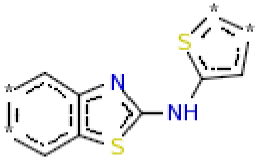<br>B7: 1474973492<br>0 out of 7 good<br>Bayesian Score: -1.544	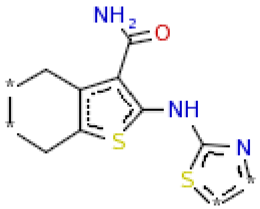<br>B8: -532506075<br>0 out of 6 good<br>Bayesian Score: -1.425	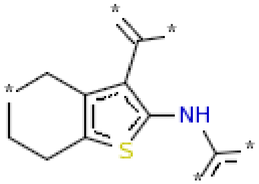<br>B9: -551147930<br>0 out of 6 good<br>Bayesian Score: -1.425	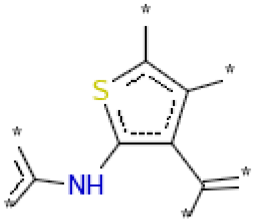<br>B10: 469153063<br>0 out of 6 good<br>Bayesian Score: -1.425

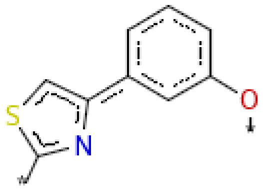<br>B11: -470831435<br>0 out of 6 good<br>Bayesian Score: -1.425	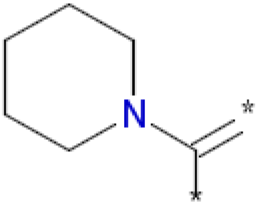<br>B12: -446103674<br>0 out of 6 good<br>Bayesian Score: -1.425	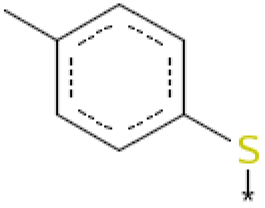<br>B13: 633795852<br>0 out of 6 good<br>Bayesian Score: -1.425	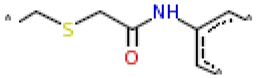<br>B14: 1415816015<br>0 out of 6 good<br>Bayesian Score: -1.425	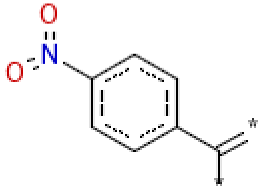<br>B15: 1786546801<br>0 out of 6 good<br>Bayesian Score: -1.425
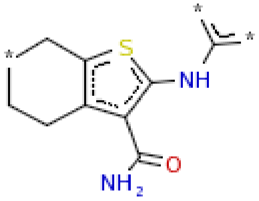<br>B16: 471397705<br>0 out of 6 good<br>Bayesian Score: -1.425	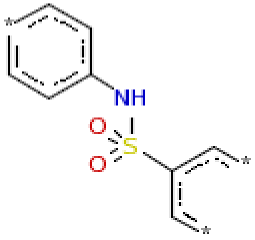<br>B17: -863010654<br>0 out of 6 good<br>Bayesian Score: -1.425	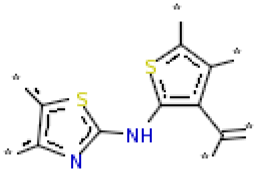<br>B18: -720826636<br>0 out of 6 good<br>Bayesian Score: -1.425	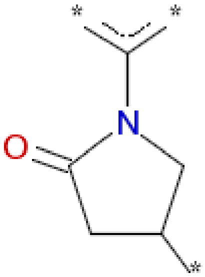<br>B19: 1867039149<br>1 out of 12 good<br>Bayesian Score: -1.297	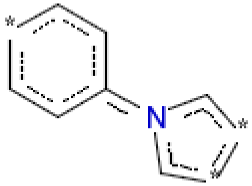<br>B20: 1889594586<br>0 out of 5 good<br>Bayesian Score: -1.289
