# Supplementary material for: Enhancing Hit Identification in Mycobacterium tuberculosis Drug Discovery Using Validated Dual-Event Bayesian Models
Source: PLoS One. 2013 May 7;8(5):e63240. doi: 10.1371/journal.pone.0063240 (PMC3647004; doi:10.1371/journal.pone.0063240)
Supplement: Figure S7 — TB kinase dose response and cytotoxicity model: good features from FCFP_6. (PDF) [file pone.0063240.s007.pdf]

Enhancing Hit Identification in *Mycobacterium tuberculosis* Drug Discovery Using Dual-Event Bayesian Models

Sean Ekins[1, 2*], Robert C. Reynolds[3,4], Scott G. Franzblau[5,], Baojie Wan[5] , Joel S. Freundlich[6,7] and Barry A. Bunin[1]

[1]Collaborative Drug Discovery, 1633 Bayshore Highway, Suite 342, Burlingame, CA 94010, USA.

[2]Collaborations in Chemistry, 5616 Hilltop Needmore Road, Fuquay-Varina, NC 27526, USA.

[3]Southern Research Institute, 2000 Ninth Avenue South, Birmingham, AL 35205, USA.

[4]Current address: University of Alabama at Birmingham, College of Arts and Sciences, Department of Chemistry, 1530 3rd Avenue South, Birmingham, Alabama 35294-1240, USA.

[5] Institute for Tuberculosis Research, University of Illinois at Chicago, Chicago, IL 60607, USA.

[6]Department of Medicine, Center for Emerging and Reemerging Pathogens, UMDNJ – New Jersey Medical School, 185 South Orange Avenue Newark, NJ 07103, USA.

[7]Department of Pharmacology & Physiology, UMDNJ – New Jersey Medical School, 185 South Orange Avenue Newark, NJ 07103, USA.

*To whom correspondence should be addressed. (e-mail: ekinssean@yahoo.com)

Running Head: Dual Event Bayesian Models

Figure S7. TB kinase dose response and cytotoxicity model: good features from FCFP_6.

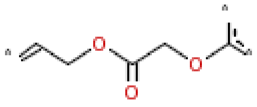<br>G1: 1670875748<br>8 out of 9 good<br>Bayesian Score: 1.373	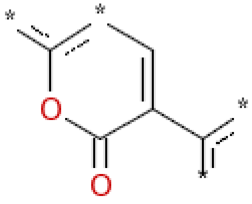<br>G2: -1235942037<br>8 out of 10 good<br>Bayesian Score: 1.312	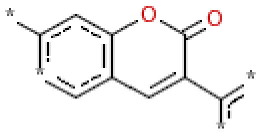<br>G3: 443956295<br>8 out of 10 good<br>Bayesian Score: 1.312	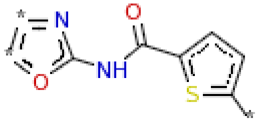<br>G4: -595817425<br>25 out of 45 good<br>Bayesian Score: 1.256	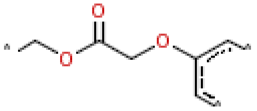<br>G5: -312381965<br>10 out of 15 good<br>Bayesian Score: 1.255
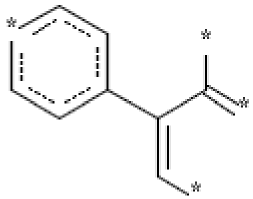<br>G6: -628297815<br>8 out of 11 good<br>Bayesian Score: 1.255	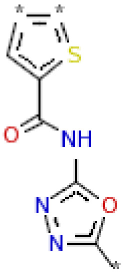<br>G7: 1158507791<br>24 out of 45 good<br>Bayesian Score: 1.217	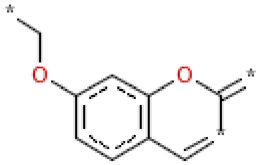<br>G8: 1953499806<br>11 out of 18 good<br>Bayesian Score: 1.215	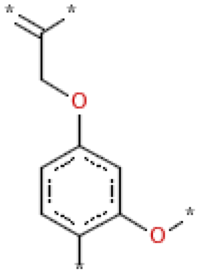<br>G9: -278994470<br>8 out of 12 good<br>Bayesian Score: 1.201	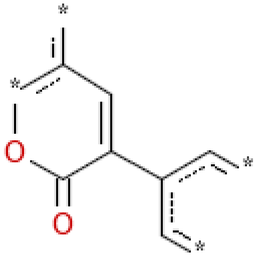<br>G10: 37329034<br>6 out of 8 good<br>Bayesian Score: 1.186

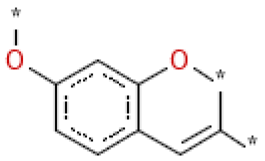<br>G11: 1193323686<br>6 out of 8 good<br>Bayesian Score: 1.186	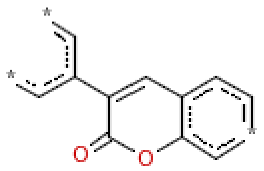<br>G12: 535094085<br>6 out of 8 good<br>Bayesian Score: 1.186	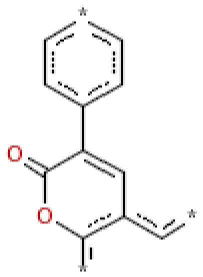<br>G13: -1631111526<br>6 out of 8 good<br>Bayesian Score: 1.186	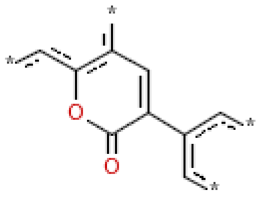<br>G14: 671951664<br>6 out of 8 good<br>Bayesian Score: 1.186	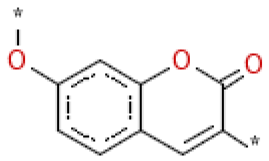<br>G15: 731843914<br>6 out of 8 good<br>Bayesian Score: 1.186
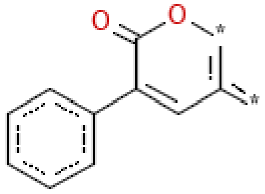<br>G16: -298028947<br>5 out of 6 good<br>Bayesian Score: 1.174	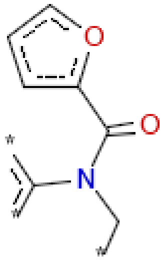<br>G17: 1662717112<br>14 out of 26 good<br>Bayesian Score: 1.160	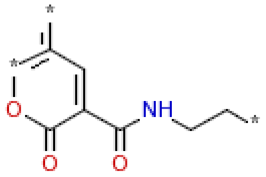<br>G18: 1393924964<br>4 out of 4 good<br>Bayesian Score: 1.159	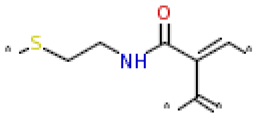<br>G19: 1768758742<br>4 out of 4 good<br>Bayesian Score: 1.159	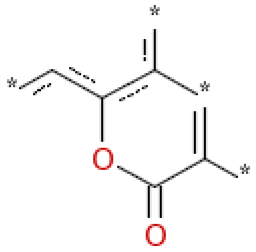<br>G20: -1979033238<br>18 out of 36 good<br>Bayesian Score: 1.132
