# Supplementary material for: Enhancing Hit Identification in Mycobacterium tuberculosis Drug Discovery Using Validated Dual-Event Bayesian Models
Source: PLoS One. 2013 May 7;8(5):e63240. doi: 10.1371/journal.pone.0063240 (PMC3647004; doi:10.1371/journal.pone.0063240)
Supplement: Figure S8 — TB kinase dose response and cytotoxicity model: bad features from FCFP_6. (PDF) [file pone.0063240.s008.pdf]

Enhancing Hit Identification in *Mycobacterium tuberculosis* Drug Discovery Using Dual-Event Bayesian Models

Sean Ekins[1, 2*], Robert C. Reynolds[3,4], Scott G. Franzblau[5,], Baojie Wan[5] , Joel S. Freundlich[6,7] and Barry A. Bunin[1]

[1]Collaborative Drug Discovery, 1633 Bayshore Highway, Suite 342, Burlingame, CA 94010, USA.

[2]Collaborations in Chemistry, 5616 Hilltop Needmore Road, Fuquay-Varina, NC 27526, USA.

[3]Southern Research Institute, 2000 Ninth Avenue South, Birmingham, AL 35205, USA.

[4]Current address: University of Alabama at Birmingham, College of Arts and Sciences, Department of Chemistry, 1530 3rd Avenue South, Birmingham, Alabama 35294-1240, USA.

[5] Institute for Tuberculosis Research, University of Illinois at Chicago, Chicago, IL 60607, USA.

[6]Department of Medicine, Center for Emerging and Reemerging Pathogens, UMDNJ – New Jersey Medical School, 185 South Orange Avenue Newark, NJ 07103, USA.

[7]Department of Pharmacology & Physiology, UMDNJ – New Jersey Medical School, 185 South Orange Avenue Newark, NJ 07103, USA.

*To whom correspondence should be addressed. (e-mail: ekinssean@yahoo.com)

Running Head: Dual Event Bayesian Models

Figure S8. TB kinase dose response and cytotoxicity model: bad features from FCFP_6.

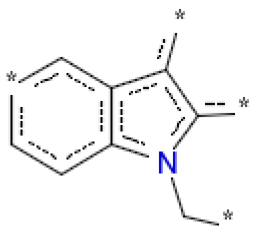<br>B1: -898059030<br>0 out of 42 good<br>Bayesian Score: -1.943	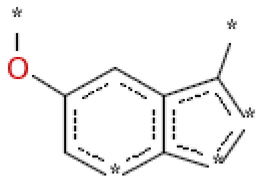<br>B2: 1674955425<br>0 out of 42 good<br>Bayesian Score: -1.943	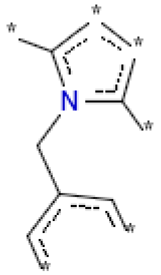<br>B3: 17443674<br>0 out of 41 good<br>Bayesian Score: -1.922	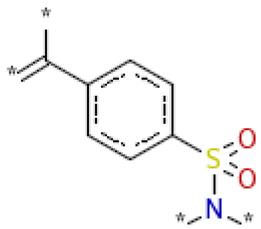<br>B4: 1790750500<br>0 out of 38 good<br>Bayesian Score: -1.858	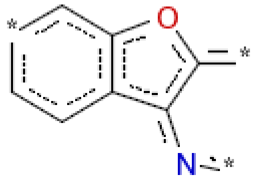<br>B5: 1526366520<br>0 out of 37 good<br>Bayesian Score: -1.835
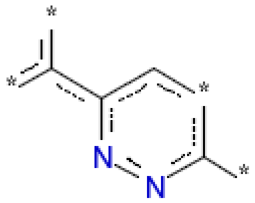<br>B6: 1964502305<br>0 out of 37 good<br>Bayesian Score: -1.835	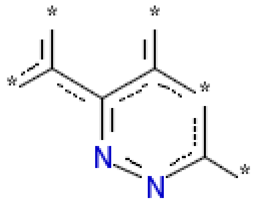<br>B7: 1087307120<br>0 out of 37 good<br>Bayesian Score: -1.835	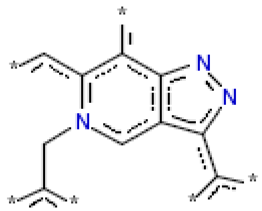<br>B8: 1813774889<br>0 out of 36 good<br>Bayesian Score: -1.812	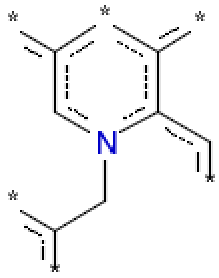<br>B9: -1731186150<br>0 out of 36 good<br>Bayesian Score: -1.812	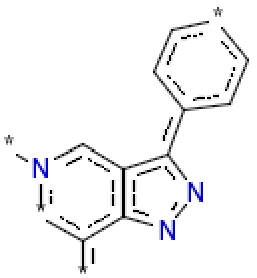<br>B10: -1236091207<br>0 out of 36 good<br>Bayesian Score: -1.812

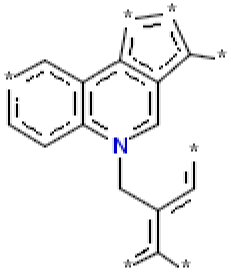<br>B11: -2024954534<br>0 out of 36 good<br>Bayesian Score: -1.812	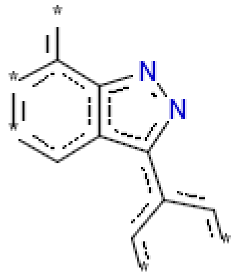<br>B12: -437082084<br>0 out of 36 good<br>Bayesian Score: -1.812	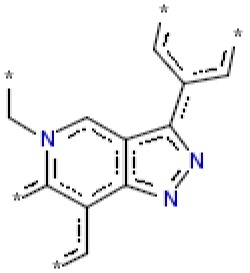<br>B13: -1528325941<br>0 out of 36 good<br>Bayesian Score: -1.812	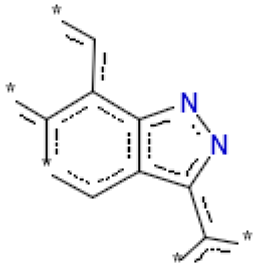<br>B14: -438280287<br>0 out of 36 good<br>Bayesian Score: -1.812	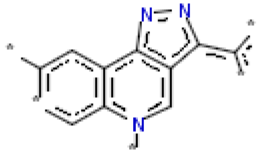<br>B15: 2056975573<br>0 out of 36 good<br>Bayesian Score: -1.812
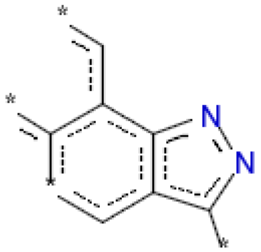<br>B16: 267940428<br>0 out of 36 good<br>Bayesian Score: -1.812	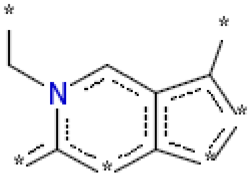<br>B17: 2101570543<br>0 out of 36 good<br>Bayesian Score: -1.812	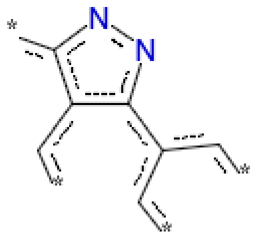<br>B18: 789148918<br>0 out of 36 good<br>Bayesian Score: -1.812	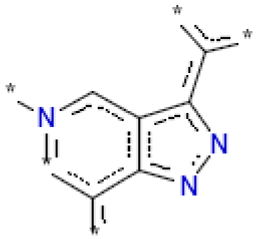<br>B19: -1227636663<br>0 out of 36 good<br>Bayesian Score: -1.812	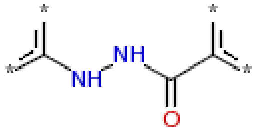<br>B20: 1935427586<br>0 out of 34 good<br>Bayesian Score: -1.765
