# Supplementary material for: Enhancing Hit Identification in Mycobacterium tuberculosis Drug Discovery Using Validated Dual-Event Bayesian Models
Source: PLoS One. 2013 May 7;8(5):e63240. doi: 10.1371/journal.pone.0063240 (PMC3647004; doi:10.1371/journal.pone.0063240)

Enhancing Hit Identification in *Mycobacterium tuberculosis* Drug Discovery Using Dual-Event Bayesian Models

Sean Ekins[1, 2*], Robert C. Reynolds[3,4], Scott G. Franzblau[5,], Baojie Wan[5] , Joel S. Freundlich[6,7] and Barry A. Bunin[1]

[1]Collaborative Drug Discovery, 1633 Bayshore Highway, Suite 342, Burlingame, CA 94010, USA.

[2]Collaborations in Chemistry, 5616 Hilltop Needmore Road, Fuquay-Varina, NC 27526, USA.

[3]Southern Research Institute, 2000 Ninth Avenue South, Birmingham, AL 35205, USA.

[4]Current address: University of Alabama at Birmingham, College of Arts and Sciences, Department of Chemistry, 1530 3rd Avenue South, Birmingham, Alabama 35294-1240, USA.

[5] Institute for Tuberculosis Research, University of Illinois at Chicago, Chicago, IL 60607, USA.

[6]Department of Medicine, Center for Emerging and Reemerging Pathogens, UMDNJ – New Jersey Medical School, 185 South Orange Avenue Newark, NJ 07103, USA.

[7]Department of Pharmacology & Physiology, UMDNJ – New Jersey Medical School, 185 South Orange Avenue Newark, NJ 07103, USA.

*To whom correspondence should be addressed. (e-mail: ekinssean@yahoo.com)

Running Head: Dual Event Bayesian Models

Figure S**9.** Results for the 194 compounds tested in the Selleckchem kinase library screened for whole-cell TB activity with Bayesian models. Random rate is based on the empirical HTS hit rate; MLSMR is based on the MLSMR dose response and cytotoxicity model; CB2 is based on the CB2 dose response and cytotoxicity model. Kinase is based on the MLSMR dose response and cytotoxicity model. Best curve is based on a 100% hit rate.

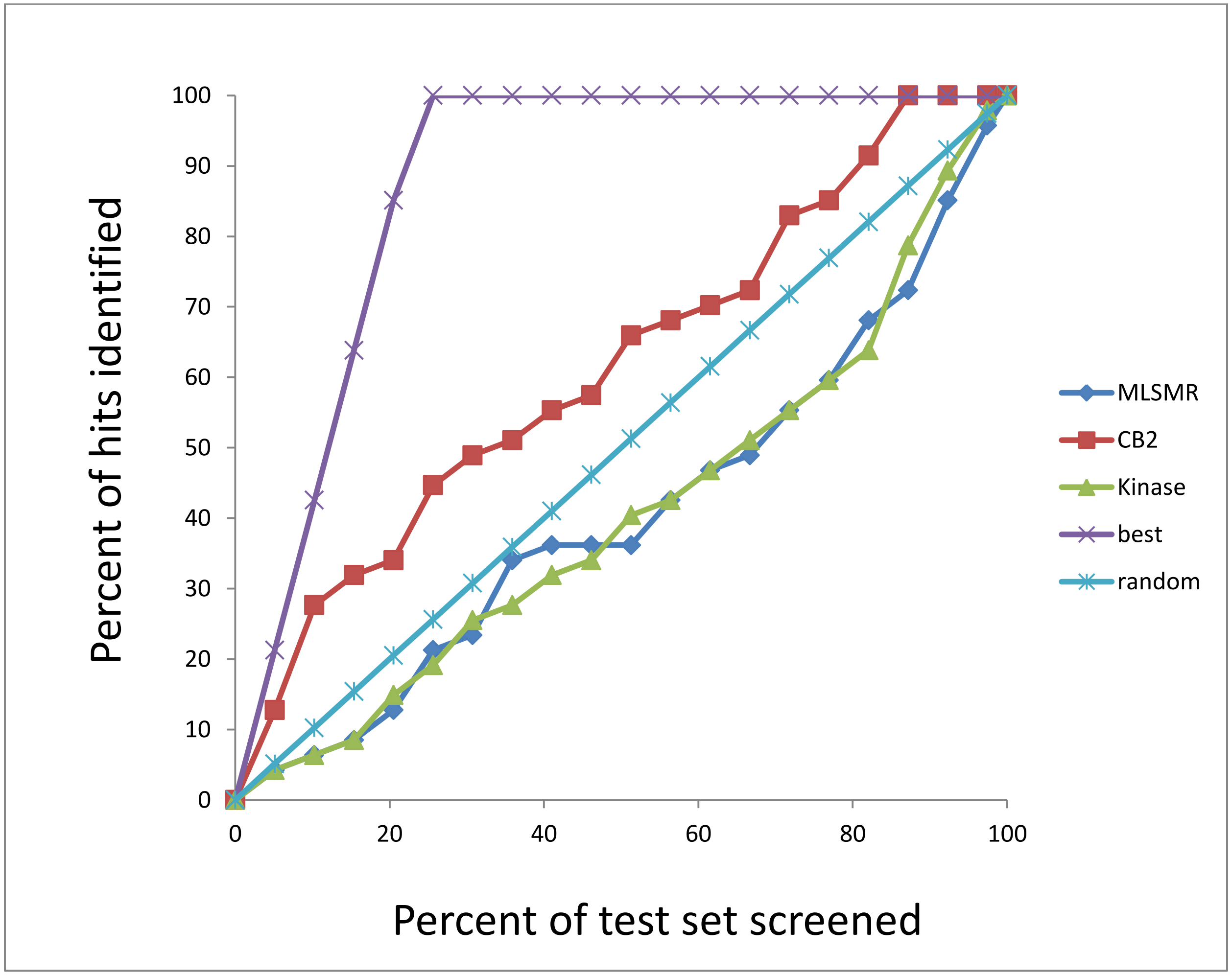

Supplement: Figure S9 — Results for the 194 compounds tested in the Selleckchem kinase library screened for whole-cell TB activity with Bayesian models. Random rate is based on the empirical HTS hit rate; MLSMR is based on the MLSMR dose response and cytotoxicity model; CB2 is based on the CB2 dose response and cytotoxicity model [32]. Kinase is based on the MLSMR dose response and cytotoxicity model. Best curve is based on a 100% hit rate. (PDF) [file pone.0063240.s009.pdf]
