# Supplementary material for: Enhancing Hit Identification in Mycobacterium tuberculosis Drug Discovery Using Validated Dual-Event Bayesian Models
Source: PLoS One. 2013 May 7;8(5):e63240. doi: 10.1371/journal.pone.0063240 (PMC3647004; doi:10.1371/journal.pone.0063240)
Supplement: Table S1 — Mean (SD) leave one out and leave out 50%×100 cross validation of previously published Bayesian models (ROC = receiver operator characteristic) – data from [24] . (PDF) [file pone.0063240.s010.pdf]

Enhancing Hit Identification in *Mycobacterium tuberculosis* Drug Discovery Using Dual-Event Bayesian Models

Sean Ekins[1, 2*], Robert C. Reynolds[3,4], Scott G. Franzblau[5,], Baojie Wan[5], Joel S. Freundlich[6,7] and Barry A. Bunin[1]

[1]Collaborative Drug Discovery, 1633 Bayshore Highway, Suite 342, Burlingame, CA 94010, USA.

[2]Collaborations in Chemistry, 5616 Hilltop Needmore Road, Fuquay-Varina, NC 27526, USA.

[3]Southern Research Institute, 2000 Ninth Avenue South, Birmingham, AL 35205, USA.

[4]Current address: University of Alabama at Birmingham, College of Arts and Sciences, Department of Chemistry, 1530 3rd Avenue South, Birmingham, Alabama 35294-1240, USA.

[5] Institute for Tuberculosis Research, University of Illinois at Chicago, Chicago, IL 60607, USA.

[6]Department of Medicine, Center for Emerging and Reemerging Pathogens, UMDNJ – New Jersey Medical School, 185 South Orange Avenue Newark, NJ 07103, USA.

[7]Department of Pharmacology & Physiology, UMDNJ – New Jersey Medical School, 185 South Orange Avenue Newark, NJ 07103, USA.

*To whom correspondence should be addressed. (e-mail: ekinssean@yahoo.com)

Running Head: Dual Event Bayesian Models

Table S1. Mean (SD) leave one out and leave out 50% x 100 cross validation of previously published Bayesian models (ROC =receiver operator characteristic) – data from [22]

**Dataset (number of molecules)**	**Leave one out ROC**	**Leave out 50% x 100 External ROC Score**	**Leave out 50% x 100Internal ROC Score**	**Leave out 50% x 100 Concordance**	**Leave out 50% x 100 Specificity**	**Leave out 50% x 100 Sensitivity**
MLSMR All single point screen (N = 220,463)	0.88	0.86 (0)	0.86 (0)	78.56 (1.86)	78.59(1.94)	77.13 (2.26)
MLSMR dose response set (N = 2273)	0.78	0.73 (0.01)	0.75 (0.01)	66.85 (4.06)	67.21 (7.05)	65.47 (7.96)
