# Supplementary material for: Enhancing Hit Identification in Mycobacterium tuberculosis Drug Discovery Using Validated Dual-Event Bayesian Models
Source: PLoS One. 2013 May 7;8(5):e63240. doi: 10.1371/journal.pone.0063240 (PMC3647004; doi:10.1371/journal.pone.0063240)
Supplement: Table S3 — SRI hits from Selleckchem picked using Bayesian dose response and cytotoxicity models (MLSMR, CB2 [32] and Kinase). More positive numbers from the Bayesian models suggest likely activity. SI is the selectivity index calculated by dividing the CC50 by either the MIC MABA or MIC LORA. Bold values have an SI greater than 10. Numbers in parentheses are the maximal similarity of the compound to members of the training set for the respective model. (DOCX) [file pone.0063240.s012.docx]

**Enhancing Hit Identification in *Mycobacterium tuberculosis* Drug Discovery Using Dual-Event Bayesian Models**

Sean Ekins^1, 2*^, Robert C. Reynolds^3,4^, Scott G. Franzblau^5,^, Baojie Wan^5^ , Joel S. Freundlich^6,7^ and Barry A. Bunin^1^

^1^Collaborative Drug Discovery, 1633 Bayshore Highway, Suite 342, Burlingame, CA 94010, USA.

## ^2^Collaborations in Chemistry, 5616 Hilltop Needmore Road, Fuquay-Varina, NC 27526, USA.

^3^Southern Research Institute, 2000 Ninth Avenue South, Birmingham, AL 35205, USA. ^4^Current address: University of Alabama at Birmingham, College of Arts and Sciences, Department of Chemistry, 1530 3^rd^ Avenue South, Birmingham, Alabama 35294-1240, USA.

^5^ Institute for Tuberculosis Research, University of Illinois at Chicago, Chicago, IL 60607, USA.

^6^Department of Medicine, Center for Emerging and Reemerging Pathogens, UMDNJ – New Jersey Medical School, 185 South Orange Avenue Newark, NJ 07103, USA.

^7^Department of Pharmacology & Physiology, UMDNJ – New Jersey Medical School, 185 South Orange Avenue Newark, NJ 07103, USA.

^*^To whom correspondence should be addressed. (e-mail: ekinssean@yahoo.com)

**Running Head**: Dual Event Bayesian Models

**Table S3.** SRI hits from Selleckchem picked using Bayesian dose response and cytotoxicity models (MLSMR, CB2 [32] and Kinase). More positive numbers from the Bayesian models suggest likely activity. SI is the selectivity index calculated by dividing the CC_50_ by either the MIC MABA or MIC LORA. Bold values have an SI greater than 10. Numbers in parentheses are the maximal similarity of the compound to members of the training set for the respective model.

| **Name** | **Structure** | **Inhibition %  MABA at 50 μg/ml** | **MIC MABA (μg/ml)** | **MIC LORA (μg/ml)** | **CC_50_ Vero (μg/ml)** | **SI**  **MABA** | **SI LORA** | **MLSMR** | **CB2** | **Kinase** |
| --- | --- | --- | --- | --- | --- | --- | --- | --- | --- | --- |
| BIBW2992(Tovok) |    \|  \| \| --- \| | 94 | 68.6 | 44.0 | 37.6 | 0.5 | 0.8 | -17.72 (0.35) | **4.46 (0.47)** | -18.48 (0.38) |
| Bosutinib(SKI-606) |    \|  \| \| --- \| | 98 | 37.7 | 42.5 | 41.8 | 1.1 | 1 | -10.89 (0.40) | **3.00 (0.43)** | -21.10 (0.32) |
| Cediranib(AZD2171) |    \|  \| \| --- \| | 100 | 23.6 | 34.2 | 32.8 | 1.4 | 0.9 | -23.67 (0.38) | -1.95 (0.37) | -19.02 (0.40) |
| CI-1033(Canertinib) |    \|  \| \| --- \| | 99 | 49.9 | 45.9 | 45.8 | 0.9 | 1.0 | -22.78 (0.38) | **4.88 (0.45)** | -21.97 (0.37) |
| Lapatinib Ditosylate |    \|  \| \| --- \| | 98 | 44.6 | 27.4 | 80.4 | 1.8 | 2.9 | -16.26 (0.40) | **2.56 (0.45)** | -21.15 (0.40) |
| Sorafenib Tosylate |    \|  \| \| --- \| | 99 | 48.0 | 35.2 | 19.9 | 0.4 | 0.6 | -19.42 (0.5) | **1.15 (0.54)** | -7.98 (0.4) |
| Vandetanib |    \|  \| \| --- \| | 96 | 79.8 | > 100 | 29.9 | 0.4 | 0.3 | -16.16 (0.42) | **3.76 (0.54)** | -22.43 (0.41) |
| BMS-599626 |    \|  \| \| --- \| | 98 | 62.2 | 70.2 | 64.3 | 1.0 | 0.9 | -3.40 (0.37) | **2.12 (0.36)** | -16.12 (0.35) |
| PF-2341066 |    \|  \| \| --- \| | 101 | 24.9 | 43.8 | 54.1 | 2.2 | 1.2 | **1.48 (0.32)** | **1.83 (0.33)** | -5.48 (0.30) |
| NVP-TAE684 |    \|  \| \| --- \| | 100 | 11.2 | 17.4 | 39.3 | 3.5 | 2.2 | -10.8 (0.40) | **1.24 (0.43)** | -19.52 (0.36) |
| XL880(GSK1363089) |    \|  \| \| --- \| | 101 | 5.9 | 5.3 | 34.5 | 5.8 | 6.5 | -26.76 (0.48) | -0.77 (0.45) | -25.38 (0.41) |
| WZ3146 |    \|  \| \| --- \| | 97 | 49.3 | 76.5 | 33.0 | 0.7 | 0.4 | -19.64 (0.44) | -1.89 (0.48) | -20.75 (0.36) |
| WZ4002 |    \|  \| \| --- \| | 99 | 45.6 | 42.3 | 35.8 | 0.8 | 0.8 | -18.72 (0.43) | -1.44 (0.46) | -19.68 (0.38) |
| BAY 73-4506(Regorafenib) |    \|  \| \| --- \| | 96 | 47.6 | 25.0 | 29.74 | 0.6 | 1.2 | -17.05 (0.52) | -0.52 (0.54) | -5.56 (0.41) |
| WZ8040 |    \|  \| \| --- \| | 100 | 25.4 | 30.2 | 42.6 | 1.7 | 1.4 | -20.65 (0.43) | -1.75 (0.48) | -20.56 (0.37) |
| Pelitinib |    \|  \| \| --- \| | 95 | 77.8 | 19.3 | 40.0 | 0.5 | 2.1 | -13.66 (0.40) | **2.73 (0.41)** | -19.14 (0.42) |
| AEE788 |    \|  \| \| --- \| | 100 | 47.5 | 48.4 | 40.3 | 0.8 | 0.8 | -19.30 (0.39) | **3.63 (0.34)** | -12.46 (0.29) |
| AP24534 |    \|  \| \| --- \| | 100 | 12.5 | 23.2 | 36.4 | 2.9 | 1.6 | -23.85 (0.47) | -3.88 (0.39) | -11.13 (0.38) |
| WP1130 |    \|  \| \| --- \| | 101 | 89.5 | 89.9 | 20.3 | 0.2 | 0.2 | -6.01 (0.48) | 0.15 (0.39) | **3.30 (0.35)** |
| PCI-32765 |    \|  \| \| --- \| | 93 | 49.1 | 47.7 | 40.1 | 0.8 | 0.8 | -10.73 (0.33) | -2.66 (0.32) | -11.44 (0.31) |
| GSK1838705A |    \|  \| \| --- \| | 99 | 47.7 | 50.0 | 68.8 | 1.4 | 1.4 | -6.31 (0.36) | **2.83 (0.35)** | -19.65 (0.33) |
| NVP-ADW742 |    \|  \| \| --- \| | 97 | 24.1 | 22.7 | 29.2 | 1.2 | 1.3 | -21.38 (0.34) | -1.17 (0.46) | -19.45 (0.30) |
| BI 2536 |    \|  \| \| --- \| | 97 | 49.4 | 47.8 | 40.9 | 0.8 | 0.8 | -3.20 (0.35) | **3.26 (0.38)** | -17.36 (0.32) |
| GSK461364 |    \|  \| \| --- \| | 99 | 48.4 | 49.9 | 49.8 | 1.0 | 1.0 | -24.24 (0.36) | -0.09 (0.33) | -10.81 (0.33) |
| HMN-214 |    \|  \| \| --- \| | 99 | > 100 | > 100 | >100 | 1 | 1 | **-0.96 (0.41)** | -0.39 (0.43) | -0.91 (0.39) |
| ENMD-2076 | \|  \| \| --- \| | 100 | 24.6 | 47.7 | 49.3 | 2.0 | 1.0 | -14.45 (0.36) | -3.72 (0.32) | -13.83 (0.3) |
| BS-181 hydrochloride |    \|  \| \| --- \| | 91 | 86.9 | 41.8 | 70.6 | 0.8 | 1.7 | -3.83 (0.38) | -1.83 (0.33) | -5.86 (0.34) |
| PD0332991 |    \|  \| \| --- \| | 95 | > 100 | > 100 | >100 | 1 | 1 | **6.66 (0.30)** | **-2.71 (0.35)** | -10.25 (0.30) |
| AZD7762 |    \|  \| \| --- \| | 93 | 77.2 | > 100 | 74.96 | 1.0 | 0.7 | -6.36 (0.45) | **0.71 (0.50)** | -9.27 (0.38) |
| Hesperadin |    \|  \| \| --- \| | 98 | 49.2 | 49.6 | >100 | 2.0 | 2.0 | -26.51 (0.40) | -0.46 (0.45) | -15.45 (0.37) |
| SNS-314 Mesylate |    \|  \| \| --- \| | 93 | 82.6 | > 100 | 39.8 | 0.5 | 0.4 | -12.97 (0.42) | **1.22 (0.41)** | -16.54 (0.46) |
| VX-680 |    \|  \| \| --- \| | 98 | 44.2 | > 100 | 45.2 | 1.0 | 0.4 | -21.44 (0.38) | -7.57 (0.40) | -18.73 (0.30) |
| ZM-447439 |    \|  \| \| --- \| | 97 | 95.9 | 89.3 | 74.2 | 0.8 | 0.8 | -21.79 (0.47) | **5.95 (0.43)** | -28.75 (0.50) |
| AT7867 |    \|  \| \| --- \| | 101 | 45.8 | 47.0 | 42.3 | 0.9 | 0.9 | -6.20 (0.46) | 1.34 (0.35) | -4.31 (0.34) |
| Deforolimus(MK-8669) |    \|  \| \| --- \| | 92 | 99.9 | > 100 | >100 | 1 | 1 | **6.60 (0.29)** | **1.49 (0.21)** | -4.98 (0.19) |
| RAF265 |    \|  \| \| --- \| | 100 | 47.0 | > 100 | 41.8 | 0.9 | 0.4 | -18.69 (0.36) | **2.83 (0.36)** | -12.61 (0.37) |
| LY2228820 |    \|  \| \| --- \| | 99 | 43.6 | 37.4 | 41.5 | 0.9 | 1.1 | -13.28 (0.34) | -1.89 (0.33) | -4.11 (0.30) |
| U0126-EtOH |    \|  \| \| --- \| | 98 | 52.2 | 94.7 | 68.4 | 1.3 | 0.7 | -5.66 (0.40) | 0.23 (0.41) | -7.47 (0.45) |
| AZD5438 |    \|  \| \| --- \| | 95 | > 100 | > 100 | >100 | 1 | 1 | -2.65 (0.44) | **3.02 (0.53)** | -14.18 (0.39) |
| CCT128930 |    \|  \| \| --- \| | 91 | > 100 | > 100 | 85 | 0.8 | 0.8 | -9.65 (0.34) | -3.22 (0.34) | -6.91 (0.32) |
| TG101209 |    \|  \| \| --- \| | 98 | 60.4 | 81.1 | 20.7 | 0.3 | 0.2 | -12.46 (0.51) | **0.99 (0.50)** | -23.71  (0.42) |
| MK-2206 | \| 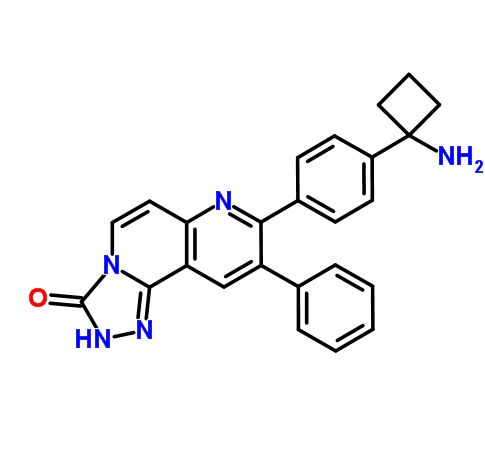 \| \| --- \| | 100 | 46.5 | 45.6 | 31.6 | 0.7 | 0.7 | -7.35 (0.36) | -3.55 (0.31) | -5.76 (0.33) |
| NVP-BSK805 | 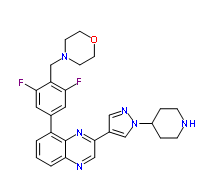   \|  \| \| --- \| | 95 | 85.9 | > 100 | 43.5 | 0.5 | 0.4 | -15.16 (0.39) | **0.94 (0.35)** | -15.36 (0.32) |
| BI6727(Volasertib) |    \|  \| \| --- \| | 96 | 97.9 | 88.1 | 40.1 | 0.4 | 0.4 | -10.00 (0.33) | **2.54 (0.35)** | -19.82 (0.29) |
| LY2603618(IC-83) |    \|  \| \| --- \| | 100 | 48.4 | 47.9 | 42.8 | 0.9 | 0.9 | -1.97 (0.39) | **1.36 (0.40)** | -3.64 (0.34) |
| PP-121 |  | 98 | 94 | 99.8 | >100 | 1.1 | 1.0 | -3.23 (0.26) | -2.66 (0.27) | -6.03 (0.24) |
| AS-604850 |  | 90 | >100 | >100 | 21.7 | 0.2 | 0.2 | -5.65 (0.49) | -1.81 (0.38) | **6.09 (0.56)** |
