# Supplementary material for: Enhancing Hit Identification in Mycobacterium tuberculosis Drug Discovery Using Validated Dual-Event Bayesian Models
Source: PLoS One. 2013 May 7;8(5):e63240. doi: 10.1371/journal.pone.0063240 (PMC3647004; doi:10.1371/journal.pone.0063240)
Supplement: Table S4 — Additional follow up compounds for the pyrazolo[1,5- a ]pyrimidine core. SI is the selectivity index calculated by dividing the CC50 by either the MIC MABA or MIC LORA. Bold values have an SI greater than 10. (DOCX) [file pone.0063240.s013.docx]

**Enhancing Hit Identification in *Mycobacterium tuberculosis* Drug Discovery Using Dual-Event Bayesian Models**

Sean Ekins^1, 2*^, Robert C. Reynolds^3,4^, Scott G. Franzblau^5,^, Baojie Wan^5^ , Joel S. Freundlich^6,7^ and Barry A. Bunin^1^

^1^Collaborative Drug Discovery, 1633 Bayshore Highway, Suite 342, Burlingame, CA 94010, USA.

## ^2^Collaborations in Chemistry, 5616 Hilltop Needmore Road, Fuquay-Varina, NC 27526, USA.

^3^Southern Research Institute, 2000 Ninth Avenue South, Birmingham, AL 35205, USA. ^4^Current address: University of Alabama at Birmingham, College of Arts and Sciences, Department of Chemistry, 1530 3^rd^ Avenue South, Birmingham, Alabama 35294-1240, USA.

^5^ Institute for Tuberculosis Research, University of Illinois at Chicago, Chicago, IL 60607, USA.

^6^Department of Medicine, Center for Emerging and Reemerging Pathogens, UMDNJ – New Jersey Medical School, 185 South Orange Avenue Newark, NJ 07103, USA.

^7^Department of Pharmacology & Physiology, UMDNJ – New Jersey Medical School, 185 South Orange Avenue Newark, NJ 07103, USA.

^*^To whom correspondence should be addressed. (e-mail: ekinssean@yahoo.com)

**Running Head**: Dual Event Bayesian Models

**Table S4.** Additional follow up compounds for the pyrazolo[1,5-*a*]pyrimidine core. SI is the selectivity index calculated by dividing the CC_50_ by either the MIC MABA or MIC LORA. Bold values have an SI greater than 10.

|  |  |  |  |  |  |  |  | |  |  |  |  |  |
| --- | --- | --- | --- | --- | --- | --- | --- | --- | --- | --- | --- | --- | --- |
| **Asinex Number** | **Structure** | **Inhibition %  MABA at 100 μg g/ml** |  | **MIC MABA (μg/ml)** | **MIC LORA (μg/ml)** | **CC_50_ Vero (μg/ml)** | **SI**  **MABA** | **SI**  **LORA** | |  |  |  |  |
|  |  |  |  |  |  |  |  | |  |  |  |  |  |
| SYN 22269211 | 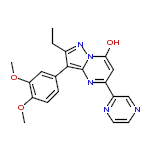   \| Image \| \| --- \| | 101 |  | 47.8 | >100 | >100 | 2.1 | | 1 |  |  |  |  |
| SYN 22269234 | *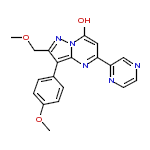*   \| *Image* \| \| --- \| | 100 |  | 49.6 | >100 | 85 | 1.7 | | 0.8 |  |  |  |  |
